# Microfluidics-based strategies for molecular diagnostics of infectious diseases

**DOI:** 10.1186/s40779-022-00374-3

**Published:** 2022-03-18

**Authors:** Xin Wang, Xian-Zhe Hong, Yi-Wei Li, Ying Li, Jie Wang, Peng Chen, Bi-Feng Liu

**Affiliations:** 1 The Key Laboratory for Biomedical Photonics of MOE at Wuhan National Laboratory for Optoelectronics-Hubei Bioinformatics & Molecular Imaging Key Laboratory, Systems Biology Theme, Department of Biomedical Engineering, College of Life Science and Technology, Huazhong University of Science and Technology, Wuhan, 430074 China; 2 State Key Laboratory of Magnetic Resonance and Atomic Molecular Physics, Wuhan National Laboratory for Optoelectronics, National Centre for Magnetic Resonance in Wuhan, Wuhan Institute of Physics and Mathematics, Innovation Academy for Precision Measurement Science and Technology, Chinese Academy of Sciences - Wuhan National Laboratory for Optoelectronics, Huazhong University of Science and Technology, Wuhan, 430071 China; 3 Department of Radiology, Canary Center at Stanford for Cancer Early Detection, School of Medicine, Stanford University, Palo Alto, CA 94304 USA

**Keywords:** Microfluidics, Molecular diagnostics, Infectious disease, Point-of-care testing (POCT), Digital assay

## Abstract

Traditional diagnostic strategies for infectious disease detection require benchtop instruments that are inappropriate for point-of-care testing (POCT). Emerging microfluidics, a highly miniaturized, automatic, and integrated technology, are a potential substitute for traditional methods in performing rapid, low-cost, accurate, and on-site diagnoses. Molecular diagnostics are widely used in microfluidic devices as the most effective approaches for pathogen detection. This review summarizes the latest advances in microfluidics-based molecular diagnostics for infectious diseases from academic perspectives and industrial outlooks. First, we introduce the typical on-chip nucleic acid processes, including sample preprocessing, amplification, and signal read-out. Then, four categories of microfluidic platforms are compared with respect to features, merits, and demerits. We further discuss application of the digital assay in absolute nucleic acid quantification. Both the classic and recent microfluidics-based commercial molecular diagnostic devices are summarized as proof of the current market status. Finally, we propose future directions for microfluidics-based infectious disease diagnosis.

## Background

Infectious diseases arise from pathogens, including bacteria, viruses, and parasites, with a global distribution. Unlike other diseases, pathogens rapidly infect and are transmitted between human and animal carriers through inoculation, air, and water media [1]. It is essential to prevent infectious diseases as a public health measure. There are three fundamental strategies for managing infectious diseases: (1) controlling the source of infection; (2) blocking transmission pathways; and (3) protecting susceptible populations. Among the fundamental strategies, control of the infectious source is considered the most crucial strategy because of convenience and low cost. Prompt diagnosis, isolation, and treatment of infected persons are essential, which require rapid, sensitive, and accurate diagnostic strategies [2]. The current diagnosis of infectious diseases usually combines clinical examinations based on signs and symptoms and laboratory tests, such as cell culture and molecular diagnostics, which require well-trained personnel, time-consuming procedures, and expensive testing equipment [3, 4]. Prevention of infectious disease outbreaks calls for rapid, low-cost, accurate, and on-site diagnosis, particularly in resource-poor areas where infectious diseases are usually prevalent and severe [5], as is treatment in the wilderness or battlefield where emergencies unpredictably occur, but medical assistance is limited [6]. In such cases, microfluidics, a technology that combines micro-electro-mechanical system technology, nanotechnology, or materials science for precise fluid manipulations [7–10], offers a new opportunity for point-of-care testing (POCT) of infectious pathogens outside of hospitals and laboratories. Microfluidic technology enables a sample- and cost-saving route for molecular diagnostics during disease outbreaks compared with traditional laborious diagnostics. The worldwide spread of corona virus disease 2019 (COVID-19) was caused by severe acute respiratory syndrome coronavirus 2 (SARS-CoV-2); as a result, the importance of microfluidics for timely prevention and control of the pandemic has again been emphasized [11–13]. Compared with traditional diagnostics, microfluidic POCT utilizes miniaturized and portable devices, ranging from benchtop analyzers to small lateral flow strips, that conduct tests nearby the sampling sites [14]. These tests are advanced for simplified or omitted sample preparation, rapid signal amplification, and sensitive signal readout, leading to a short duration and accurate results within minutes. The availability and massive production of microfluidics-based point-of-care tools have expanded their applications for cost-effective and straightforward diagnosis outside the hospital, near the patient, or even at home.

**Fig. 1:**
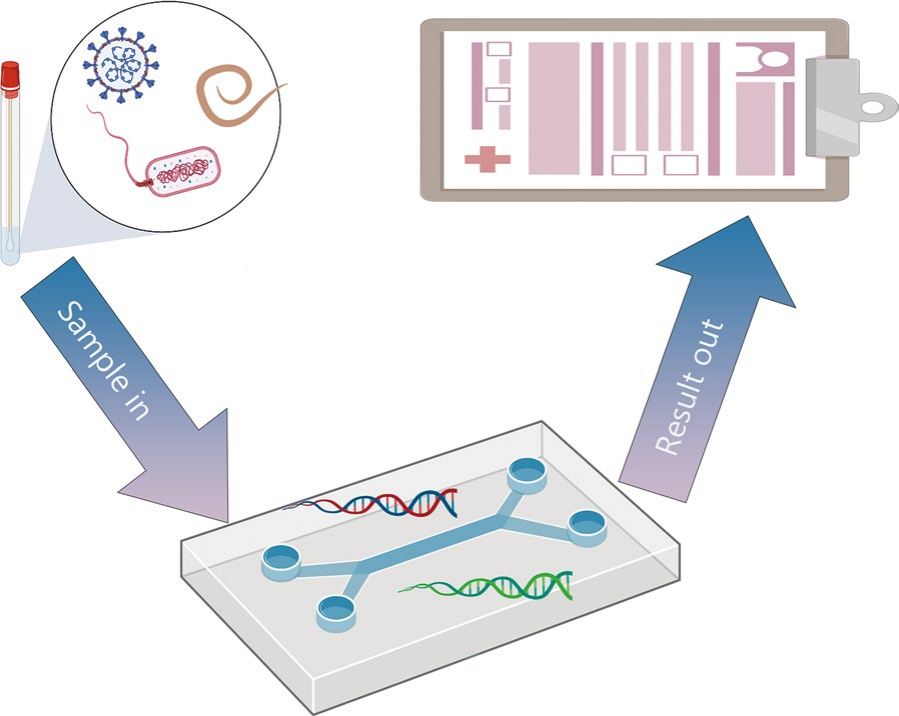
Principal diagram of microfluidics-based strategies for molecular diagnostics of infectious diseases

Among the existing strategies for diagnosing infectious diseases, molecular diagnostics are among the most sensitive methods [15, 16]. Moreover, molecular diagnostics usually serve as the gold standard method for ongoing COVID-19 detection, allowing direct detection of virus-specific RNA or DNA regions prior to onset of the immune response [17, 18]. In the current review, we present the latest advances in microfluidics-based processes for molecular diagnostics of infectious diseases, from an academic perspective to future industrial outlook (Fig. 1). We start with the three steps critical for nucleic acid testing: on-chip sample pre-processing; nucleic acid amplification; and signal read-out. We then compared various types of microfluidic platforms with their structures and functions, which showed unique features (both pros and cons). The digital nucleic acid assay is further discussed and exemplified as the third-generation technology for the absolute quantification of infectious pathogen molecules. Additionally, several typical and latest commercial POCT devices will be introduced, which display the current state of the microfluidic POCT market for molecular diagnostics. Our outlooks towards future applications will also be discussed and explained.

## On-chip nucleic acid testing

Based on the implemented functions, the modules of a microfluidic chip for nucleic acid testing can be divided into three categories (sampling, sensing, and signaling) [19]. Among these modules, the sampling module mainly realizes sample lysis and nucleic acid extraction. The sensing module primarily operates the conversion and amplification of nucleic acid signals. The signaling module achieves detection of the signal after conversion and processing by the sensing module. We will summarize different chips that can achieve the “sample in and answer out” function according to the on-chip nucleic acid testing procedure.

### Sampling module: lyse the original samples and extract nucleic acids

The foremost step of nucleic acid testing is nucleic acid extraction, which refers to the isolation of targeted nucleic acid from the original samples. Nucleic acid extraction is performed to purify nucleic acids from other molecular pollutants, ensure the integrity of the primary structure of nucleic acid molecules, and to optimize outturns. Nucleic acid extraction requires essential sample lysis and nucleic acid capture, the quality and efficiency of which have a huge impact on the research and diagnosis results. Any subtle adverse effects during extraction limit downstream detections. For example, polymerase chain reaction (PCR) and loop-mediated isothermal amplification (LAMP) approaches are inhibited by some residual organic solvents in nucleic acid extraction reagents such as ethanol and isopropanol [20]. Liquid–liquid extraction and solid-phase extraction are among the most popular modes of nucleic acid extraction [21]; however, liquid–liquid extraction on the chips is extremely limited because the reagents used in liquid–liquid extraction are corrosive to most microfluidic chips. Herein we emphasize solid-phase extraction methods based on microchips and compare the strengths and weaknesses.

#### Silicon-based strategies

Silicon is a compatible substrate material for nucleic acids because silicon is biocompatible, stable, and has easily modifiable properties [22]. Importantly, when modified by silica or other materials, this composite exhibits the characteristic of adsorbing negatively-charged nucleic acids in low pH and hypersaline conditions, while eluting with high pH and low-salt solutions. Based on this phenomenon, nucleic acids can be purified.

Silicon-based materials of various forms have been exploited for nucleic acid extraction in microfluidics, such as silica beads, powder, microfiber filters, and silica gel membranes [23–26]. Depending on the material properties, silicon-based materials can be utilized in various ways on microchips. For example, silica beads, powders, and commercial nanofilters can be simply placed into the wells or microchannels of the microfluidic chip and assist the extraction of nucleic acids from samples [27–29]. Surface-modified silica gel membranes can also be used to rapidly purify DNA from pathogens at low cost. For example, Wang et al. [30] introduced a universal and portable system by combining a denaturation bubble-mediated strand exchange amplification reaction with chitooligosaccharide-coated silica gel membranes through which 10^2^–10^8^ colony-forming units (CFU)/ml of *Vibrio parahaemolyticus* were successfully detected, and the existence of the virus was easily visualized. Powell et al. [31] then used the silicon-based microchip to detect hepatitis C virus (HCV), human immunodeficiency virus (HIV), Zika virus, and human papilloma virus multiply and automatically, in which 1.3 µl of meandering microreactors were designed to capture RNA of viruses and perform in situ amplification. In addition to these methods, surface-modified silicon micropillars play a key role in nucleic acid extraction because the geometrical dimension and modifying material properties significantly improve extraction efficiency. Chen et al. [32] proposed a microfluidic platform to extract RNA at low concentrations based on amino-coated silicon micropillars. The microfluidic device integrates micro-pillar arrays within an area of 0.25 cm^2^ on the silicon substrate to substantiate a higher extraction efficiency with high surface-to-volume ratio designs. As a benefit from this design, the microfluidic device achieves up to 95% nucleic acid extraction efficiency. These silicon-based strategies demonstrated the value of rapid isolation nucleic acids at low cost. When combined with microfluidic chips, silicon-based extraction strategies not only improve the efficiency of nucleic acid testing, but also facilitate miniaturization and integration of analytical devices [20].

#### Magnetic-based strategies

The magnetic-based isolation approach exploits magnetic particles to extract nucleic acids at the circumstance of external magnetic fields. The commonly utilized magnetic particles include silica-coated, amino-coated, and carboxyl-coated Fe_3_O_4_ or γ-Fe_2_O_3_ magnetic particles [33–36]. Compared with silicon-based, solid-phase extraction techniques, a distinct feature of the magnetic particles is ease of manipulation and control using an external magnet.

**Fig. 2:**
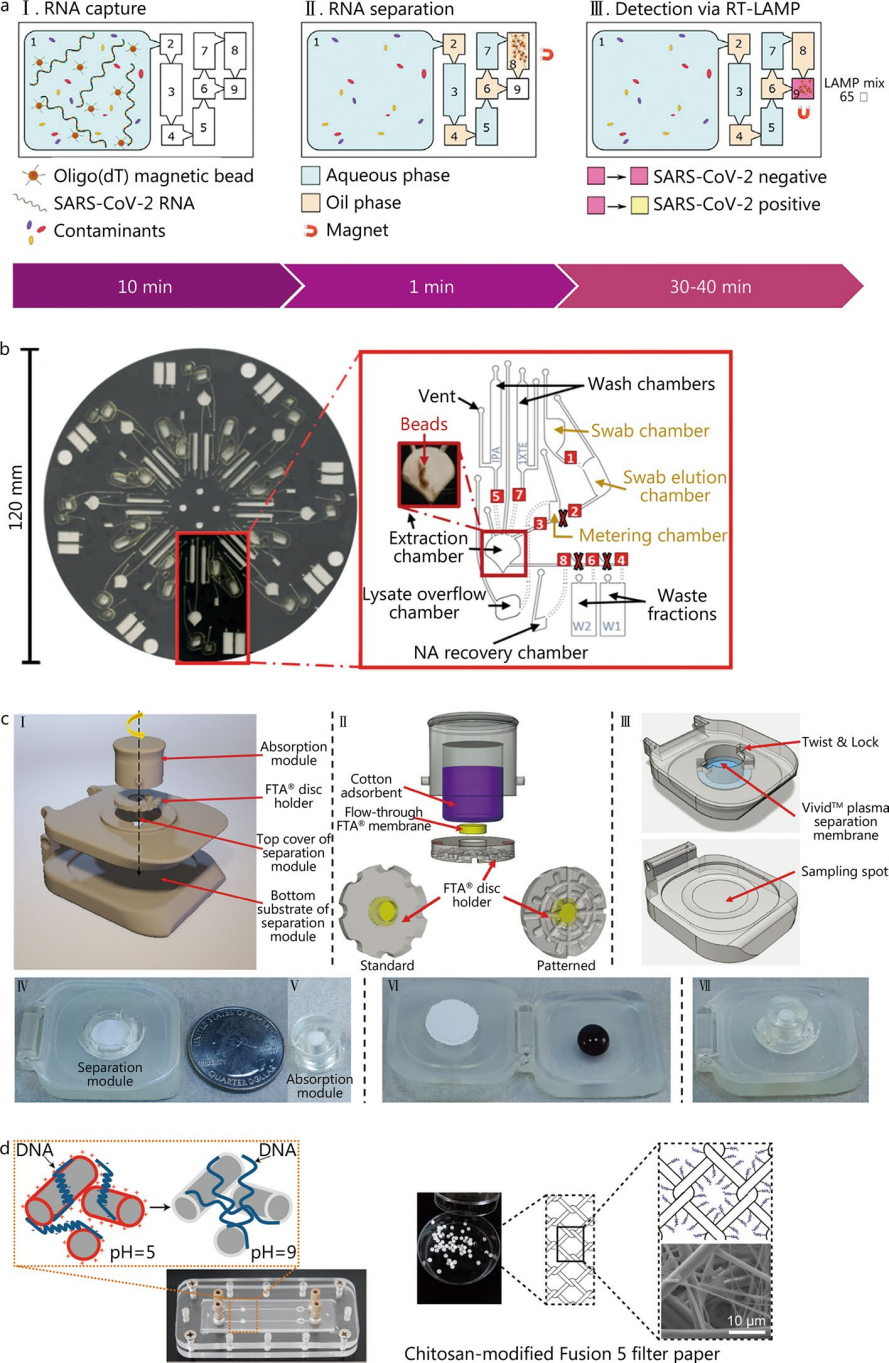
Magnetic- and porous material-based devices. **a** Conceptual scheme for the microfluidic IFAST RT-LAMP device for SARS-CoV-2 RNA detection (adapted from [38]). **b** Centrifugal microdevice for dSPE of nucleic acids from buccal swabs (adapted from [39]). **c** Self-powered integrated sample concentrator using FTA^®^ card (adapted from [50]). **d** Chitosan-modified Fusion 5 filter paper (adapted from [51]). SARS-CoV-2 severe acute respiratory syndrome coronavirus 2, RT-LAMP reverse-transcription loop-mediated isothermal amplification, FTA finders technology associates, NA nucleic acid

Utilizing the electrostatic interactions between nucleic acids and silica, nucleic acids are adsorbed to the surface of silica-encapsulated magnetic particles under hypersaline and low pH conditions, while the molecules can be eluted again under hyposaline and high pH conditions. The silica-coated magnetic beads allow for DNA extraction from large-volume samples (400 μl) with the help of magnet-guided movement [37]. As a demonstration, Rodriguez-Mateos et al. [38] used a tunable magnet to manipulate the transfer of magnetic beads in different chambers. Based on silica-coated magnetic particles, 470 copies/ml of genomic SARS-CoV-2 RNA can be extracted from wastewater samples for reverse-transcription LAMP (RT-LAMP) detection, and the answer can be read out within 1 h by the unaided eye (Fig. 2a).

The positively-charged magnetic particles are ideal for the nucleic acid phosphate backbone to attach. At a specific salt concentration, the negatively-charged nucleic acid phosphate groups can be absorbed to the surface of magnetic composite particles by positive charges. Thus, the magnetic nanoparticle with a rough surface and a high density of amino groups has been developed for nucleic acid extraction. After magnetic separation and blocking, the magnetic nanoparticles and DNA complexes can be used directly for PCR, omitting complex and time-consuming purification and elution operations [35]. The negative carboxyl-coated magnetic nanoparticles are also made to isolate nucleic acids, which are adsorbed to the surface in high concentrations of polyethylene glycol and sodium chloride solutions [36]. Utilizing these surface-modified magnetic beads, DNA extraction is compatible with downstream amplification. Dignan et al. [39] described an automatic and portable centrifugal microfluidic platform for nucleic acid pre-processing that allows in situ use by non-technical personnel. Moreover, the compatibility of the extracted DNA with LAMP, a technique ideal for point-of-care nucleic acid analysis, was further demonstrated for minimal hardware requirements and adaptability with a colorimetric assay (Fig. 2b).

The magnetic bead methods provide the possibility for automated extraction, of which some commercial automatic nucleic acid extractors exist [KingFisher; ThermoFisher (Waltham, MA, U.S.), QIAcube^®^HT; CapitalBio (Beijing, China), and Biomek^®^; Beckman (Miami, FL, U.S.)]. The advantages of magnetic beads in combination with microfluidics for automated nucleic acid extraction with high efficiency have the potential to facilitate the growth of molecular diagnostics; however, magnetic beads in combination with microfluidics are still largely dependent on complex control systems to precisely manipulate magnetic beads, which explains why prevailing commercial products are bulky and expensive, restricting the further application of magnetic beads in POCT.

#### Porous materials-based strategies

Several porous materials, such as modified nitrocellulose filter, Finders Technology Associates (FTA) cards, polyethersulfone-based filter paper, and glycan-coated materials, have also been utilized for nucleic acid detection [40–44]. Porous fibrous materials, such as fibrous papers, are first used for DNA extraction utilizing the physical entanglement of long-chain DNA molecules with the fiber. Small pores lead to strong physical constraints on DNA molecules, which has a positive effect on DNA extraction. The extraction efficiency does not satisfy the need for DNA amplification due to the varying sizes of pores of the fibrous paper [45, 46]. The FTA card, a commercial filter paper used in the forensic field, has been widely applied to other molecular diagnostics. By using cellulose filter paper impregnated with various chemicals to help lyse cellular membranes from samples, the released DNA can be protected from degradation for up to 2 years. More recently, impregnated cellulose paper has been developed for molecular testing of various pathogens, including SARS-CoV-2, leishmaniasis, and malaria [47–49]. The HIV in separated plasma is directly lysed, and viral nucleic acids are enriched by an integrated, flow-through FTA^®^ membrane in the concentrator, which enables nucleic acid preparation with high efficiency [50] (Fig. 2c). The main challenge for nucleic acid testing using FTA cards is that the chemicals, such as guanidine and isopropanol, will inhibit subsequent amplification reactions. To solve the problem, chitosan-modified Fusion 5 filter paper was developed for high-efficiency nucleic acid extraction by combining the strengths of both leveraging the physical entanglement of DNA molecules with the fiber filter paper and the electrostatic adsorption of DNA to the chitosan-modified filter fibers [51] (Fig. 2d). Similarly, Zhu et al. [52] demonstrated a chitosan-modified capillary assist, a microfluidic-based in situ PCR method, to rapidly extract and detect Zika virus RNA. Based on the features of the chitosan with pH-responsive “on and off” switches, nucleic acids can be adsorbed/desorbed in a lysate/PCR mixture environment, respectively.

As described, these strategies incorporate the strengths of different solid-phase materials and increase the performance of nucleic acid extraction in microfluidics. In practical applications, extensive use of these materials is not economical, while using the materials for proper processing or surface modification of common materials can also maintain their functions. Thus, it is believed that cost can be decreased by implementing these strategies after pilot studies.

### Sensing module: convert and amplify nucleic acid signals

Nucleic acid testing on microfluidic platforms often uses small sample volumes (< 100 µl), therefore requires amplification of the target nucleic acids with specific probes for conversion to a signal that is convenient for downstream detection (optical, electrical, and magnetic) [53, 54]. Nucleic acid amplification in microfluidics can also speed up the reaction, optimize the limit of detection, lower the sample demand, and increase the detection accuracy [55, 56]. Recently, with the achievement of fast and accurate detection, various nucleic acid amplification methods, including PCR and some isothermal amplification reactions, have been applied in microfluidics. This section will summarize those promising techniques based on microfluidic systems for nucleic acid testing.

#### PCR

**Fig. 3:**
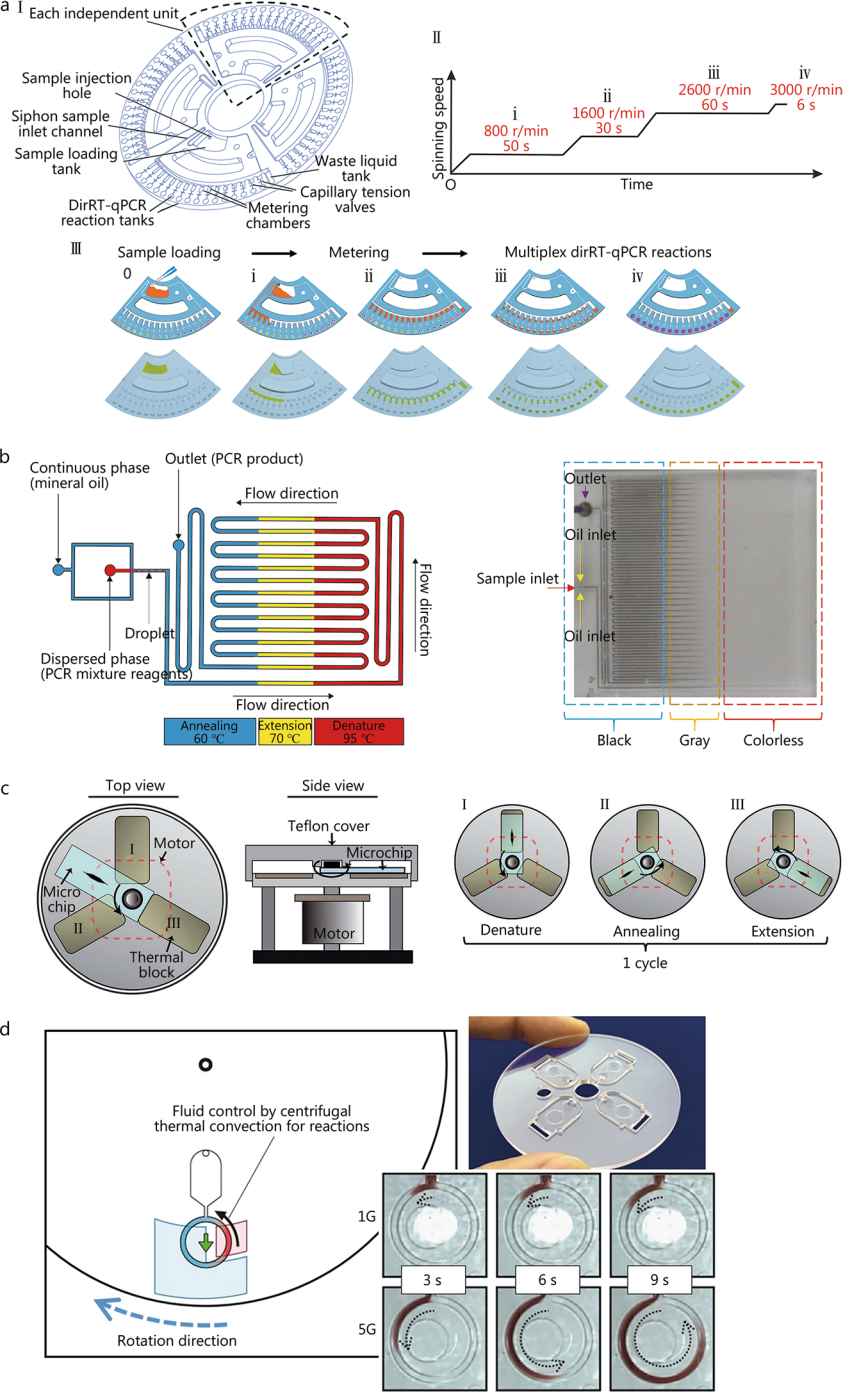
Applying PCR in microfluidics. **a** Schematics of the dirRT-qPCR in the microfluidic platform (adapted from [60]). **b** Schematics of long serpentine channel based continuous flow PCR microchip (adapted from [62]). **c** Schematic illustration of a Rotary PCR Genetic Analyzer, which consists of a microchip, three heat blocks, and a stepper motor (adapted from [63]). **d** Schematic diagram of centrifugal-assisted thermal convection PCR and devices (adapted from [64]). DirRT-qPCR direct reverse-transcription quantitative polymerase chain reaction

PCR is a simulation of the DNA replication procedure from organisms, the theory of which is detailed elsewhere and thus will not be discussed herein. PCR can amplify very few target DNA/RNA at an exponential rate, thus making PCR a powerful tool to detect nucleic acids rapidly. In recent decades, many portable microfluidic devices equipped with thermal circulation systems to perform PCR have been developed to satisfy the needs of point-of-care diagnosis [57, 58]. According to different temperature control methods, on-chip PCR can be divided into four types (traditional, continuous-flow, spatially-switched, and convective PCR) [59]. For example, Ji et al. [60] established the direct reverse-transcription quantitative PCR (RT-qPCR) assay on a self-designed microfluidic platform to multiply detect SARS-CoV-2, and influenza A and B viruses in pharyngeal swab samples (Fig. 3a). Park et al. [61] established a simple pathogen analytic chip by integrating the film-based PCR, electrode, and polydimethylsiloxane-based finger-actuated microfluidic modules. Nevertheless, both works exemplify the common disadvantage of traditional PCR. Thermal cycling is necessary for PCR, which restricts the further miniaturization for the device and shorter testing time.

The development of microfluidics-based continuous flow and spatially-switched PCR is essential to solve this problem. Utilizing a long serpentine channel or short straight channel, continuous flow PCR can achieve rapid amplification by actively pushing reagents with a pump outside of chips to three pre-heated zones in sequence and circularly. The operation successfully avoids the transition stage between different reaction temperatures, which significantly reduces the testing time [62] (Fig. 3b). In another study, Jung et al. [63] proposed a novel Rotary PCR Genetic Analyzer to perform the ultrafast and multiple reverse-transcription PCR in combination with the features of the stationary and flow-through PCR (Fig. 3c). The PCR microchip will rotate through three thermal blocks with different temperatures for nucleic acid amplification, as follows: I. block at 94 °C for denaturation; II. block at 58 °C for annealing; and III. block at 72 °C for the extension.

Through capillary tubes and loops, or even thin disks, convective PCR can rapidly amplify nucleic acids with naturally induced free thermal convection without an external pump. For instance, a cycle olefin polymer microfluidic platform was developed on a fabricated rotating heater stage utilizing a centrifugation-assisted thermal cycle in a ring-structured microchannel for PCR [64] (Fig. 3d). The reaction solution is driven by thermal convection and continuously exchanged high/low temperatures in the ring-structured microchannel. The whole amplification process can be finished in 10 min and the limit of detection goes to 70.5 pg/channel.

As expected, rapid PCR is a powerful tool for both fully-integrated “sample-to-answer” molecular diagnostic systems and multiplex analysis systems. With rapid PCR, the time spent on detecting SARS-CoV-2 is significantly decreased, which helps to control the COVID-19 pandemic efficiently.

#### Isothermal amplification

A complex thermocycler is required for PCR, which is inappropriate for POCT. Recently, isothermal amplification methods have been applied to microfluidics, including but not limited to LAMP, recombinase polymerase amplification (RPA), and nucleic acid sequence-based amplification [65–68]. With these technologies, nucleic acids are amplified at a constant temperature, thus promoting portable POCT devices for molecular diagnostics with low cost and high sensitivity.

**Fig. 4:**
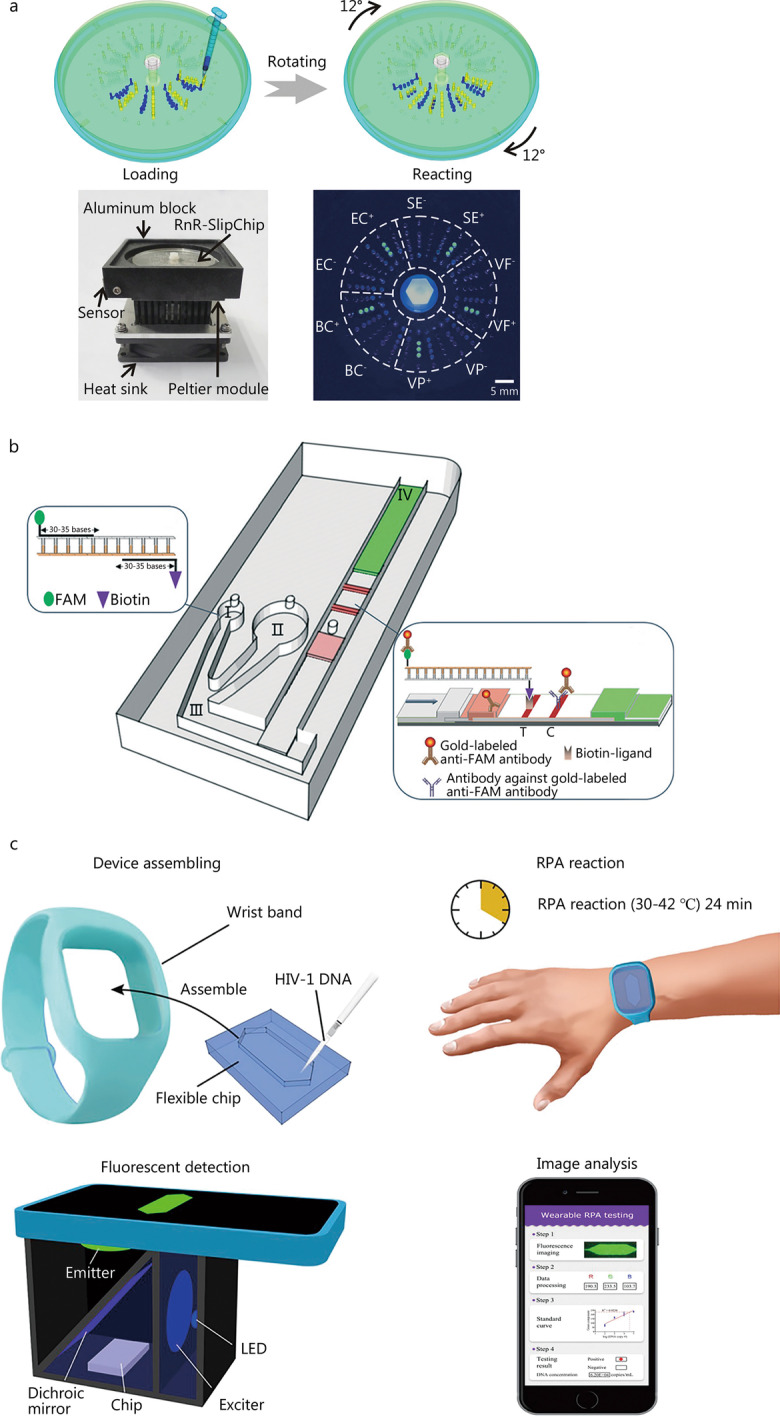
Isothermal amplification in point-of-care testing (POCT). **a** Design and fabrication of the rotate and react SlipChip. After plasma bonding, a screw-nut suite was used to assemble the upper and lower chips to form the final chip (adapted from [76]). **b** Schematic illustration of the MI-IF-RPA system for COVID-19 detection (adapted from [85]). **c** Schematic of wearable RPA testing for rapid detection of HIV-1 DNA (adapted from [86]). *SE* *Salmonella enterica*, *VF* *Vibrio fluvialis*, *VP* *Vibrio parahaemolyticus*, *BC* *Bacillus cereus*, *EC* *Escherichia coli*, FAM carboxyfluorescein, HIV human immunodeficiency virus, RPA recombinase polymerase amplification, LED light emitting diode, MI-IF-RPA microfluidic-integrated lateral flow recombinase polymerase amplification

High-throughput microfluidics-based LAMP analysis enables multiplex detection of infectious diseases [42, 69–71]. In combination with centrifugal microfluidic systems, LAMP can further promote the automation of nucleic acid detection [69, 72–75]. A rotate and react SlipChip was developed to visually detect multiple bacteria in parallel by LAMP [76] (Fig. 4a). With optimized LAMP in the assay, the fluorescent signal-to-noise ratio is approximately fivefold, and the limit of detection reached 7.2 copies/μl genomic DNA. Moreover, the existence of five common digestive bacterial pathogens, including *Bacillus cereus*, *Escherichia coli*, *Salmonella enterica*, *Vibrio fluvialis* and *Vibrio parahaemolyticus*, were visualized based on the method in < 60 min.

The advantages of LAMP in microfluidics include, but are not limited to rapid reaction and miniaturized detection. Yet, due to the reaction temperature during LAMP (approximately 70 °C), aerosols are inevitably produced, which results in a high rate of false-positive results. Detection specificity, primer design, and temperature control also need to be optimized for LAMP. Moreover, chip designs that implement multiple target detection on one chip are of significant value and should be developed. Furthermore, LAMP is suitable for multiple target detection integrated into one chip, which is of great significance, but still has a large room for growth.

RPA can partially reduce the high false-positive rates of LAMP because the relatively low reaction temperature (approximately 37 °C) causes a relatively small evaporation problem [77]. In the RPA system, two opposing primers initiate the DNA synthesis by combining with the recombinant enzymes and the amplification can be completed within 10 min [78–81]. Therefore, the entire process of RPA is much faster than PCR or LAMP. Microfluidic technology has been demonstrated to further improve the velocity and accuracy of RPA in recent years [82–84]. For example, Liu et al. [85] developed a microfluidic-integrated lateral flow recombinase polymerase amplification assay to rapidly and sensitively detect SARS-CoV-2, integrating the reverse-transcription RPA (RT-RPA) and a universal lateral flow dipstick detection system into a single microfluidic system (Fig. 4b). The assay can be finished in approximately 30 min with a 1 copy/μl or 30 copies/sample limit of detection. A wearable microfluidic device was developed by Kong et al. [86] for rapid and straightforward detection of HIV-1 DNA through RPA utilizing body temperature and a cellphone-based fluorescence detection system (Fig. 4c). The wearable RPA testing can detect target sequences at 100 copies/ml within 24 min, showing great potential for rapid diagnosis of HIV-1-infected infants in resource-limited areas.

RPA based on microfluidics has witnessed rapid advances; however, the cost from chip fabrication and reaction consumption is too high and is supposed to be lowered to increase the accessibility of the technique. In addition, the high sensitivity of RPA may influence the amplification of non-specific products, especially when contamination exists. These limitations may affect the application of RPA in microfluidic systems and deserve further optimization. Well-designed primers and probes for different targets are also required to increase the feasibility of RPA-based microfluidic strategies in POCT.

#### Clustered regularly interspaced short palindromic repeats (CRISPR)-based methods for nucleic acid testing

Cas13 and Cas12a have the ability to cut nucleic acids indiscriminately, and thus can be developed as detection and diagnostic tools. Cas13 and Cas12a are activated when binding the target DNA or RNA, respectively. Once activated, the proteins then start to cut other nucleic acids nearby, after which the guide RNA that targets pathogen-specific nucleic acids can cut off a quenched fluorescent probe and unleash fluorescence. Based on the theory, Kellner et al. [87] developed a Cas13-based method [Specific High-sensitivity Enzymatic Reporter UnLOCKING (SHERLOCK)], while Broughton et al. [88] developed another Cas12a-based method [DNA Endonuclease Targeted CRISPR Trans Reporter (DETECR)].

In recent years, various CRISPR-based nucleic acid assays have emerged [89, 90]. Traditional CRISPR-based methods are usually time-consuming and labor-intensive because of multiple procedures encompassing nucleic acid extraction, amplification, and CRISPR detection. The likelihood of false-positive results may be increased for exposing liquid to air. Given the above, the CRISPR-based systems are in urgent need of optimization.

**Fig. 5:**
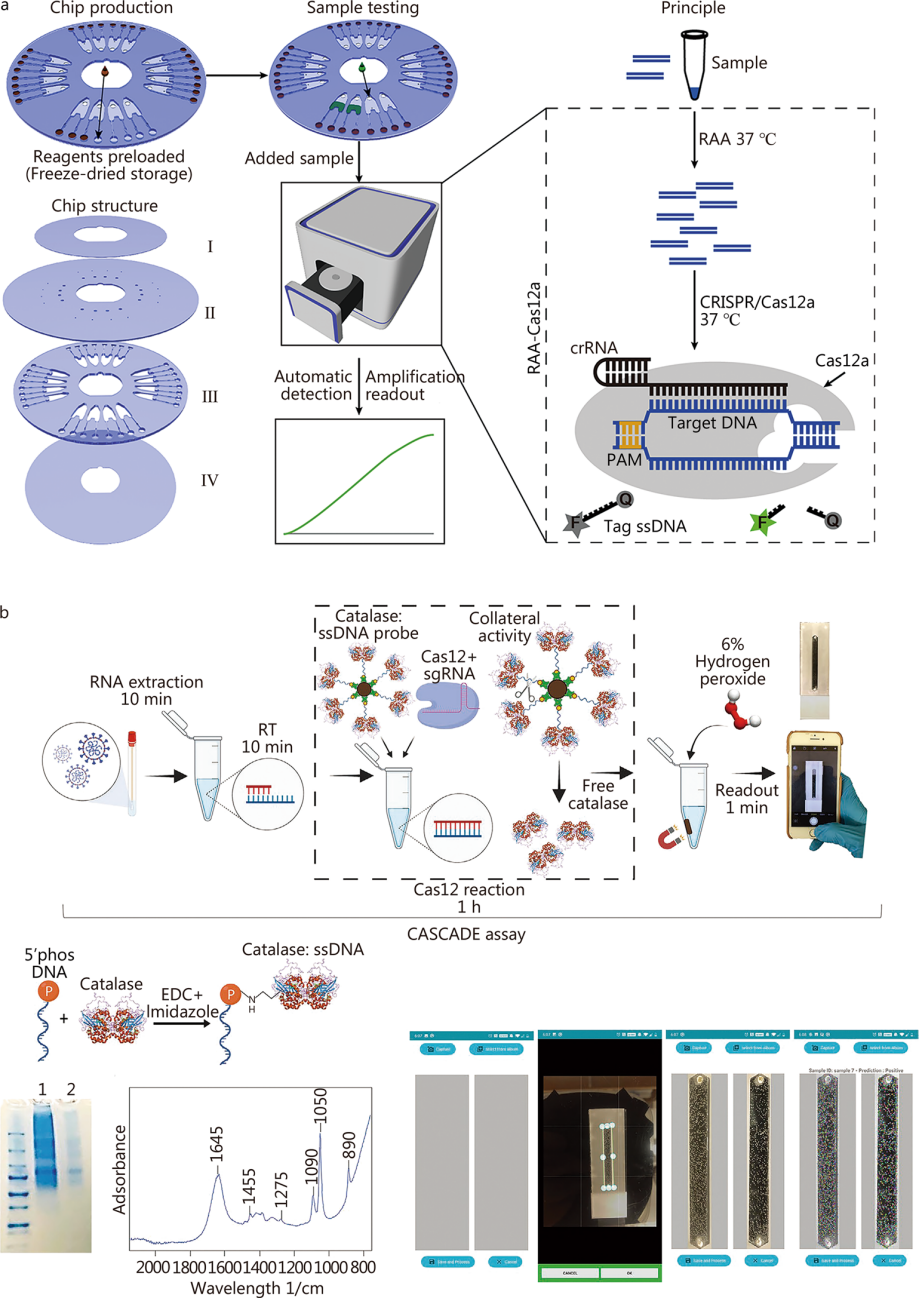
CRISPR-based methods for nucleic acid testing. **a** Integrated CRISPR-based molecular diagnostic centrifugal POCT (adapted from [92]). **b** Development of the CASCADE assay for smartphone-based SARS-CoV-2 detection (adapt from [93]). RAA recombinase-aided amplification, PAM protospacer adjacent motif, CRISPR clustered regularly interspaced short palindromic repeats, CASCADE Cellphone-Based Amplification-Free System with CRISPR/CAS-dependent enzymatic, EDC 1-ethyl-3-[3-dimethylaminopropyl] carbodiimide hydrochloride

A pneumatically-controlled microfluidic platform that can run 24 assays in parallel was designed for CRISPR-Cas12a and CRISPR-Cas13a detection applications [91]. The system is equipped with a fluorescence detection device, thus can automatically detect femtomolar DNA and RNA samples bypassing nucleic acid amplification. Chen et al. [92] integrated recombinase-aided amplification with CRISPR-Cas12a system in centrifugal microfluidics (Fig. 5a). This work overcomes the difficulty in integrating these two processes because Cas12a can digest the template DNA and inhibit the amplification process. In addition, Chen et al. [92] further pre-stored reaction reagents into centrifugal microfluidics to complete the whole process automatically. In another work, Silva et al. [93] developed an amplification-free CRISPR/Cas12a- and smartphone-based diagnostic method to detect SARS-CoV-2 (Fig. 5b). This assay is referred to as a cellphone-based amplification-free system with CRISPR/Cas-dependent enzyme, relying on smartphone imaging of a catalase-generated gas bubble signal in a microfluidic channel. Less than 50 copies/µl nucleic acids can be sensitively detected without pre-amplification and the full process from sample inlet to signal readout takes only 71 min.

### Signaling module: detect signals processed by sensing module

As the final step of the nucleic acid testing, signal detection directly reflects the diagnostic result and is determinative in developing efficient, sensitive, and accurate POCT. Signals can be read out through various methods, such as fluorescence-based, electrochemical, colorimetric, and magnetic-based strategies. In this section, we will introduce the principle of each approach and make a comparison for infectious disease molecular diagnostics in microfluidics.

**Fig. 6:**
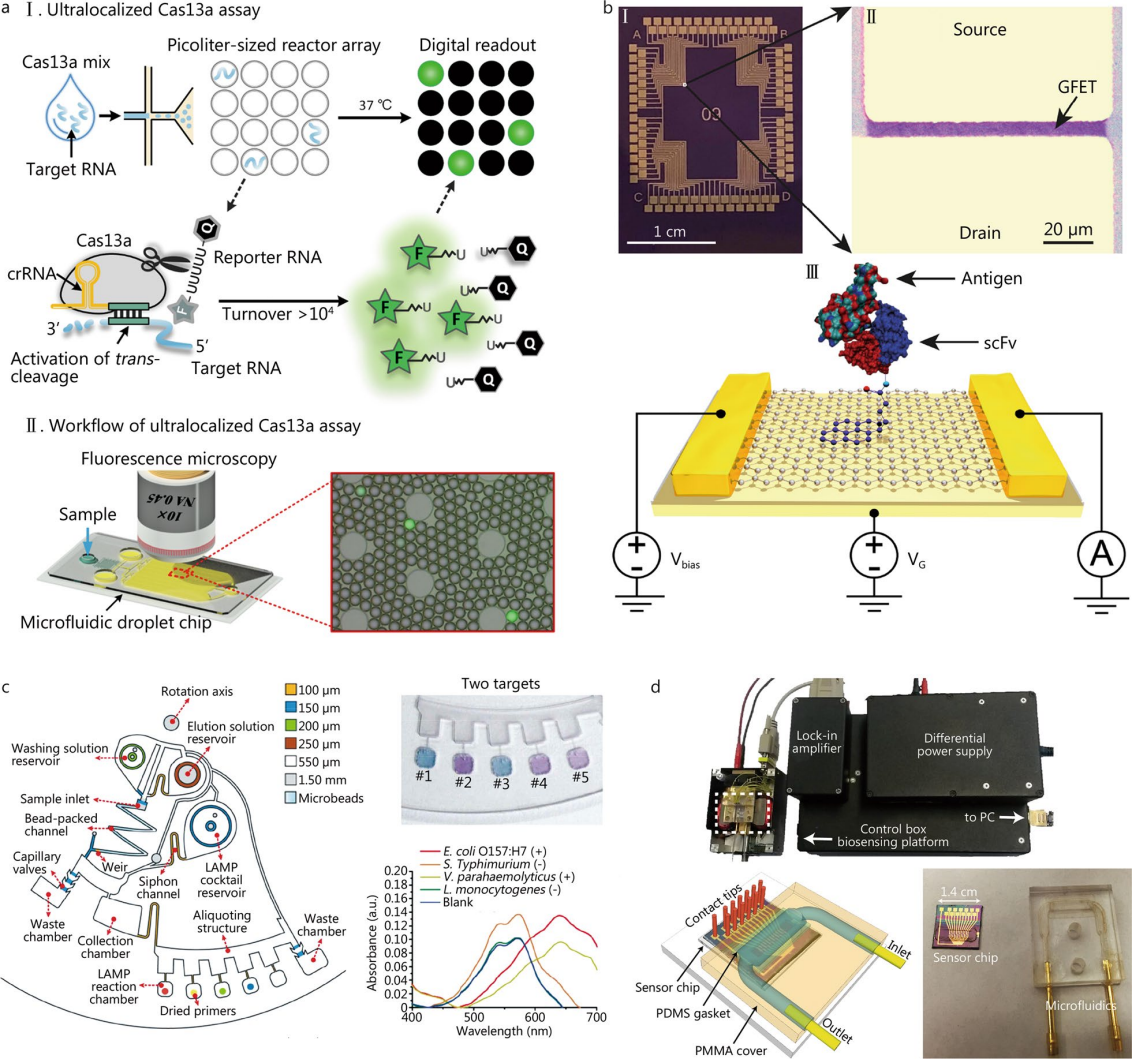
Typical approaches for signal detection. **a** Concept of ultra-localized Cas13a assay (adapted from [97]). **b** Graphene field-effect transistors nano-biosensor conjugated with Lyme GroES scFv (adapted from [98]). **c** Colorimetric read-out of multiplex detection of foodborne pathogens in the centrifugal microfluidic chip: #1 and #3 are samples with target pathogens; and #2, #4, and #5 are samples without target pathogens (adapted from [103]). **d** The magnetic tunneling junction-based biosensor comprising the platform, the integrated lock-in amplifier, the control box for signal generation/acquisition and the power supply (adapted from [106]). GFET graphene field-effect transistor, *E. coli* *Escherichia coli*, *S. typhimurium* *Salmonella typhimurium*, *V. parahaemolyticus* *Vibrio parahaemolyticus*, *L. monocytogenes* *Listeria monocytogenes*, PC personal computer, PDMS polydimethylsiloxane, PMMA polymethylmethacrylate

Fluorescence-based strategies are extensively applied to POCT to diagnose infectious diseases owing to their significant benefits of superior sensitivity, low cost, easy operation, and instant analysis [94, 95]. These strategies make use of labeling fluorophores, such as fluorescent dyes and nanomaterials, to produce detectable signals (fluorescence enhancement or quenching). This finding suggests that fluorescence-based strategies can be categorized into direct fluorescence labeling, “signal-on” and “signal-off” fluorescence detection [96]. Direct fluorescence labeling detection makes use of special fluorescent tags labeling specific ligands to generate a certain amount of fluorescence when selectively binding to the targets. For “signal-on” fluorescence detection, the quality of the fluorescence signal is positively correlated with the target quantity. The fluorescence intensity is insignificant in the absence of the target and detectable as the target is sufficient. Conversely, the fluorescence intensity of the “signal-off” fluorescence detection is negatively correlated with the target quantity, which is initially at a maximum and decreasing, while the target is enhancing. For example, by utilizing the target-dependent trans-cleavage mechanism of CRISPR-Cas13a, Tian et al. [97] developed a novel sensing strategy to detect RNA directly bypassing reverse transcription (Fig. 6a). Binding to a complementary target RNA, the CRISPR–Cas13–RNA complex can be activated, triggering collateral cleavage of a non-specific RNA reporter in trans. The fluorescently-labeled reporter [fluorophore (F)] is quenched by a quencher (Q) when intact and generates fluorescence when cleaved by the activated complex.

Electrochemical detection has advantages, such as rapid detection, easy fabrication, low cost, portability, and self-control, making it a powerful analytical method for POCT applications. Based on a graphene field-effect transistor, Gao et al. [98] developed a nano-biosensor to multiply detect antigens of Lyme disease from *Borrelia burgdorferi* bacteria, exhibiting a 2 pg/ml limit of detection (Fig. 6b).

Colorimetric assays have been applied for POCT applications, benefitting from the dominance of portability, low cost, ease of preparation, and naked eye readout. Colorimetric detection can convert the information of target nucleic acid existence to visible color change utilizing oxidation of peroxidase or peroxidase-like nanomaterials, aggregation of nanomaterials, and addition of dye indicators [99–101]. Notably, gold nanoparticles are broadly applied in colorimetric strategy establishment and have attached increasing interest to develop colorimetric POCT platforms for on-site infectious disease diagnostics because of the ability to cause fast and significant color changes [102]. Utilizing an integrated centrifugal microfluidic device [103], foodborne pathogens within a contaminated milk sample can be automatically detected down to 10 bacterial cell levels, the outcome of which can be read out by the unaided eye in 65 min (Fig. 6c).

Magnetic-based sensing methods can sensitively detect analytes by employing the magnetic materials, and have obtained a surging interest for POCT applications in recent decades. Magnetic-based sensing methods have some unique advantages, such as low-cost magnetic materials rather than expensive optics components. Even so, the detection efficiency is improved and the sample preparation time is decreased utilizing magnetic fields [104]. Moreover, magnetic-based sensing results exhibit great specificity, sensitivity, and high signal-to-noise ratio because of the insignificant magnetic background signal of biological samples [105]. A magnetic tunneling junction-based biosensor was integrated onto a portable microchip platform by Sharma et al. [106] for the multiplex detection of pathogens (Fig. 6d). The biosensor sensitively detects extracted nucleic acids below the nanomole range from pathogens.

Despite the outstanding performance of the detection methods mentioned above, drawbacks still exist. These methods were compared (Table 1), including some applications with detailed information (both advantages and disadvantages).

**Table 1:** Comparison of the detection methods for infectious diseases based on microfluidics

Detecting methods	Microfluidic systems	Amplification methods	Extracting methods	Analyte	Performance	Pros and cons	References
Fluorescence	µPAD	RCA	CNAEK	SARS-CoV-2 RNA	0.7 aM 15 min	#Superior sensitivity, low cost, easy to operate, rapid analysis *High background noise	[4]
	LOCC	LAMP	CNAEK	Virus DNA	74 copies/µl 30 min		[21]
	LOCC	RCA	Silica beads	Influenza RNA	0.5 fM 40 min		[25]
	LOAD	–	Silica beads	*E. coli* O157:H7 DNA	60% EE		[27]
	–	SEA	Silica membrane	*Vibrio parahaemolyticus* DNA	10 CFU/g		[30]
	LOCC	RT-qPCR	Silicon micropillars	Virus RNA	95% EE		[32]
	µPAD	PCR	Chitosan porous membranes	Bacteria DNA	89% EE		[40]
	–	LAMP	Microcapillary	Blood DNA	0.2 µl sample 150 min		[41]
	µPAD	RT-PCR	FTA membranes	HIV RNA	3 copies/µl 5 min		[50]
	LOAD	RT-PCR	CNAEK	SARS-CoV-2 RNA	10 copies/µl 15 min		[53]
	LOAD	RT-PCR	CNAEK	SARS-CoV-2, influenza A and B RNA	10 copies/µl 57 min		[60]
	LOAD	PCR	CNAEK	β-actin DNA	70.5 pg/channel 10 min		[64]
	LOAD	RT-LAMP	CNAEK	SARS-CoV-2 RNA	2 copies/µl 70 min		[72]
	LOAD	RT-LAMP	CNAEK	Virus RNA	10 copies/µl 40 min		[74]
	LOAD	RT-LAMP	CNAEK	SARS-CoV-2 RNA	15 copies/µl 45 min		[75]
	µPAD	RT-RPA	CNAEK	HIV RNA	5 copies/µl 45 min		[78]
	LOAD	RPA	CNAEK	*S. aureus* DNA	3 CFU/µl 60 min		[79]
Electrochemistry	–	–	Silicon nitride nano filter	*E. coli* O157:H7 DNA	–	#Rapid detection, easy to fabricate, low cost, portable and self-controlled *Unstable and susceptible	[28]
	–	–	Silicon nitride nano filter	Micro RNA	30 min		[29]
	LOCC	LAMP	Magnetic beads	Bacteria DNA	10 copies/µl 15 min		[33]
	LOCC	RT-LAMP	Magnetic beads	SARS-CoV-2 RNA	470 copies/µl 60 min		[38]
	LOCC	RT-PCR	Magnetic beads	Virus RNA	40 copies/µl 100 min		[43]
	LOCC	PCR	CNAEK	*E. coli* O157:H7 DNA	100 CFU/ml 60 min		[61]
Colorimetry	µPAD	LAMP	FTA card	Blood DNA	90 min 3 copies/µl	#Portable, low cost, easy to prepare, naked eye readout *Unable to quantitatively detect, limited sensitivity	[44]
	–	PCR	FTA card	Leishmaniasis DNA	60 min		[47]
	–	PCR	FTA card	Plasmodium DNA	3 parasites/µl 45 min		[48]
	LOAD	RT-LAMP	CNAEK	SARS-CoV-2 RNA	0.1 copy/µl 10 min		[73]
Magnetic	LOCC	–	Magnetic beads	*H. pylori* DNA	DNA purification and 40-fold pre-concentration within 7 min	#Low cost, efficient, rare sample preparation, negligible magnetic background *Hard to read out magnetic signals with miniaturized systems	[37]
	LOAD	RT-LAMP	Magnetic beads	SARS-CoV-2 RNA	8 samples in parallel 30 min		[39]

Performance includes the limit of detection, testing time (min), extraction efficiency (EE), and other characteristics (# refers to advantage and * refers to disadvantage). "–" means the information is not officially available from public data or is too complicated to be noted; *LOCC* lab on a cartridge chip, *LOAD* lab on a disc, *µPAD* microfluidic paper-based analytical device, *RCA* rolling circle amplification, *LAMP* loop-mediated isothermal amplification, *SEA* strand exchange amplification, *RT-qPCR* reverse-transcription quantitative polymerase chain reaction, *PCR* polymerase chain reaction, *RT-LAMP* reverse-transcription loop-mediated isothermal amplification, *RT-RPA* reverse-transcription recombinase polymerase amplification, *CNAEK* commercial nucleic acid extraction kits, *FTA* Finders Technology Associates, *aM* 10^–18^ mol/L, *fM* 10^–15^ mol/L, *CFU* colony-forming units, *SARS-CoV-2* severe acute respiratory syndrome coronavirus 2, *E. coli Escherichia coli*, *S. aureus Staphylococcus aureus*, *H. pylori Helicobacter pylori*

## Integrated microfluidic platforms for infectious disease diagnosis

With the development of microfluidics, micro-electro-mechanical system, nanotechnology, and materials science, the application of microfluidic chips for infectious disease detection has been promoted continuously [55, 96, 107, 108]. The miniaturized devices and precise manipulations of fluids facilitate the accuracy and economy of diagnosis. Therefore, great efforts have been made to optimize and innovate chips for further development, which leads to different microfluidic chips of various structures and functions. Herein, we briefly introduced a few common types of microfluidic platforms and compared their features (advantages and disadvantages). Furthermore, most of the examples listed below mainly focus on targeting SARS-CoV-2.

### Lab on a cartridge chip (LOCC)

LOCC is the most common micro total analysis system, in which manipulation is highly miniaturized, integrated, automated, and parallelized from sample input and preparation, flow control, and liquid detection [109, 110]. Manipulation of fluids are performed by well-designed geometries and the interplay of multiple physical effects, such as pressure gradients, capillarity, electro-kinetics, magnetic fields, and sound waves [111]. LOCC shows excellent advantages in high-throughput screens and multiple assays with fast analysis, small sample volume, low power consumption, and efficient control and manipulation; however, LOCC devices are so delicate that it is also difficult to fabricate, package, and interface, while multiplexing and reuse are of great challenge [96]. Compared with other platforms, LOCC exhibits several exclusive merits in maximum application diversity and best compatibility for technologies, while drawbacks are also obvious in the high complexity and weak reproducibility. The dependence on external pumps, which are usually huge and expensive, further constrains its usage for POCT.

During the COVID-19 outbreak, a large amount of attention has been paid to LOCC. Meanwhile, some novel chips integrated with various techniques emerged. For example, smartphones are now widely available as portable analytic devices and have great potential to integrate with LOCC. Sun et al. [21] fabricated a microfluidic chip that can multiply amplify specific nucleic acid sequences of five pathogens, including SARS-CoV-2 by LAMP, and detected them at the end of the reactions by a smartphone in 1 h. As another example, Sundah et al. [112] formed a molecular switch [catalytic amplification by the transition-state molecular switch (CATCH)], which can directly and sensitively detect SARS-CoV-2 RNA targets with a smartphone. CATCH is compatible with portable LOCC and achieves superior performance (approximately 8 RNA copies/μl; < 1 h at room temperature) [112]. In addition, some driving forces are also used in LOCC equipment for molecular diagnostics, such as vacuum, stretching, and electric fields. Kang et al. [113] demonstrated an ultrafast and real-time nano-plasmonic on-chip PCR to rapidly and quantitatively diagnose COVID-19 on site using the vacuum-driven plasmofluidic PCR chip. Li et al. [114] subsequently developed a stretching-driven microfluidic chip that realized the diagnosis of COVID-19. The platform adopted a RT-LAMP amplification system to decide whether the sample qualitatively tested positive or negative. Subsequently, Ramachandran et al. [115] achieved a proper electric field gradient utilizing isotachophoresis (ITP), a selective ionic focusing technique-to implement in microfluidics. Through ITP, target RNA within raw nasopharyngeal swab samples can be automatically purified. Then, Ramachandran et al. [115] incorporated this ITP purification with LAMP and the ITP-enhanced CRISPR assay for SARS-CoV-2 detection in approximately 35 min from both contrived and clinical nasopharyngeal swab samples. Additionally, new ideas are being launched all the time. Jadhav et al. [116] proposed a diagnostic protocol based on surface-enhanced Raman spectroscopy coupled with microfluidic devices that contain integrated microchannels functionalized with vertically-aligned aurum/argentum-coated carbon nanotubes or with disposable electrospun micro/nano-filter membranes. This device adsorbs viruses from various biological fluids/secretions, such as saliva, the nasopharynx, and tears. Therefore, the viral titer is enriched and the viruses can be accurately identified from the Raman signatures.

### Lab on a disc (LOAD)

LOAD is a centrifugal microfluidic platform, in which all the processes are controlled by the frequency protocol of a rotating micro-structured substrate [110]. The LOAD device is characterized by utilizing centrifugal forces as significant driving forces. Fluids are also controlled by capillary, Euler, and Coriolis forces. With a centrifugal unit, assays are conducted by sequential liquid operations from radial inward-to-outward positions, leaving out extra external tubes, pumps, actuators, and active valves. Briefly, the sole control method eases manipulation. The forces on liquids at the same distance from the center of the LOAD and in the identical microfluidic channel are equal, making the repeats of channel structure possible. Thus, it is easier and more economical to design and fabricate LOAD devices than conventional LOCC ones, while reactions are highly independent and parallelized; however, because of the high mechanical strength of the centrifugal equipment, the available materials of chips are limited and small volumes are hard to be performed. Simultaneously, most LOAD devices are for one-time use only, which is high-cost for large-scale assays [96, 117–119].

LOAD is regarded as one of the most promising microfluidic devices and has received great attention from researchers and manufacturers in recent decades. As a result, LOAD has been widely accepted and utilized in molecular diagnostics of infectious pathogens [120–124], especially during the outbreak of COVID-19. For example, at the end of 2020 Ji et al. [60] showed the direct RT-qPCR assay to detect SARS-CoV-2, and influenza A and B viral infections in parallel from pharyngeal swab samples rapidly and automatically. Then, Xiong et al. [74] presented a disk-like microfluidic platform integrated with LAMP for rapid, accurate, and simultaneous detection of seven human respiratory coronaviruses, including SARS-CoV-2, within 40 min. In early 2021, de Oliveira et al. [73] displayed a polystyrene-toner centrifugal microfluidic chip manually controlled by a fidget spinner for molecular diagnostics of COVID-19 by RT-LAMP. Subsequently, Dignan et al. [39] revealed an automated, portable, centrifugal microdevice to purify SARS-CoV-2 RNA directly from buccal swab cuttings. Xiong et al. [53] presented a small-volume rotating microfluidic fluorescence chip-integrated aerosol SARS-CoV-2 sampling system with a detection limit of 10 copies/μl and the shortest cycle threshold of 15 min. Soares et al. [75] recently reported the development of an integrated modular centrifugal microfluidic platform to detect SARS-CoV-2 RNA by LAMP directly from heat-inactivated nasopharyngeal swab samples. These examples demonstrate a huge advantage in applying LOAD in molecular diagnostics of COVID-19 and good prospects for growth.

### Microfluidic paper-based analytical devices (μPADs)

In 1945, Müller and Clegg [125] first introduced the microfluidic channel on paper by using filter paper and paraffin. In 2007 the Whitesides group [126] created the first functional paper platform to test protein and glucose. The paper has become an ideal substrate for microfluidics. Papers have intrinsic properties, such as a hydrophilic and porous structure, excellent biocompatibility, lightweight, flexibility, fold ability, low cost, ease of use, and availability. Classic μPADs are composed of hydrophilic/hydrophobic structures built on paper substrates. Based on the three-dimension structure, μPADs can be classified into two dimensional (2D) and three dimensional (3D) μPADs. 2D μPADs are produced by patterning hydrophobic borders to form microfluidic channels, while 3D μPADs are usually made from stacking of 2D microfluidic paper layers, and sometimes by paper folding, slip techniques, open channels, and 3D-printing [96]. Aqueous solutions or biological fluids on μPADs are mainly controlled by capillary forces without external power sources, thus facilitating reagent pre-storage, sample manipulation, and multiplex detection. Nevertheless, precise control of flow and multiple assays are blocked while lacking detection speed, sensitivity, and reusability [96, 127–130].

As an extraordinary microfluidic platform, μPADs have been greatly promoted and developed for molecular diagnostics of infectious diseases, such as HCV, HIV, and SARS-CoV-2 [131, 132]. To detect HCV selectively and sensitively, Teengam et al. [133] developed a novel fluorescent paper-based biosensor employing a highly specific pyrrolidinyl peptide nucleic acid probe. The nucleic acid was covalently immobilized onto partially oxidized cellulose paper through reductive alkylation between the amine and aldehyde groups, while the detection was based on fluorescence. The signals can be read out by a custom-made portable fluorescent camera gadget combined with a cellphone camera. Subsequently, Lu et al. [134] constructed a flexible paper-based electrode based on a nickel metal–organic framework composite/aurum nanoparticles/carbon nanotubes/polyvinyl alcohol for target HIV DNA detection by DNA hybridization using methylene blue as a redox indicator. Recently, Chowdury et al. [135] proposed a hypothetical design of a μPADs point-of-care platform for COVID-19 analyte detection using unprocessed patient-derived saliva, combined with LAMP and a handheld image acquisition technique.

### Lateral flow assay (LFA) chips

Lateral flow tests drive liquids by capillary forces and control fluid movement by the wettability and characteristic structure of the porous or micro-structured substrate. The lateral flow device consists of sample, conjugate, incubation and detection, and absorbent pads. Nucleic acid molecules in a LFA recognize specific conjugates pre-stored on the conjugate pad and combined as complexes. When the fluid pass through the incubation and detection pad, the complexes will be captured by the capture molecules located on the test and control line, showing results that can be read directly by the unaided eye. Typically, LFA can be completed in 2–15 min, which is faster than traditional assays. Due to its special mechanism, LFA requires few operations and omits extra equipment, which is user-friendly. It is convenient for fabrication and miniaturization, while paper-based substrate also has a low cost. Yet, it is only for qualitative analysis and has great difficulty for quantitative detection, while multiplexing capability and throughput are so limited that only one kind of nucleic acid that is sufficient can be tested at a time [96, 110, 127].

Even though most applications of LFA are focused on immunoassay, applying LFA for molecular diagnostics in microfluidic chips is also efficient and popular [136]. Using hepatitis B virus, HIV, and SARS-CoV-2 LFA as examples, Gong et al. [137] presented an upconversion nanoparticle-based LFA platform and demonstrated the universality of this miniaturized and portable platform by sensitively and quantitatively detecting several targets, such as hepatitis B virus nucleic acids. Furthermore, Fu et al. [138] showed a novel surface-enhanced Raman spectroscopy-based LFA for the quantitative analysis of low concentration HIV-1 DNA. To rapidly and sensitively detect SARS-CoV-2, Liu et al. [85] developed the microfluidic-integrated lateral flow RPA assay, combining the RT-RPA and a universal lateral flow dipstick detection system into a single microfluidic system.

The applications of different microfluidic platforms are varied in specific research, taking advantage of the platform capabilities and merits. LOCC is the most inclusive platform for application diversity and technology compatibility with the maximum development possibilities because of available valves, pumps, and channels. Therefore, we hope and suggest that the most novel research be conducted in LOCC as a first attempt and that conditions are optimized. In addition, more efficient and accurate approaches are expected to be discovered and utilized in the system. LOAD succeeded in precisely controlling liquids from available LOCC devices and showed unique advantages in the solo driver by centrifugal forces without an external actuator, while parallel reactions could be individual and synchronized. Thus, LOAD will be the mainstream of future microfluidic platforms with decreased artificial operations, requiring more mature and automatic techniques. The μPAD platforms integrate the advantages of both LOCC and paper material, and are suitable for inexpensive and one-time diagnosis. Therefore, future development should focus on technologies that are convenient and well-developed. Furthermore, LFA is a highly suitable for unaided eye detection, which is expected to reduce sample consumption and accelerate testing speed. The detailed comparison of the platforms is shown in Table 2.

**Table 2:** Comparison of microfluidic platforms for diagnosis

Platforms	Features compared with other platforms	Driving forces	Advantages	Disadvantages	References
LOCC	Maximum application diversity Best compatibility for technologies High complexity Weak reproducibility	Pressure gradient Capillary effects Electric fields Magnetic fields Lorentz forces Acoustic wave	High-throughput, multiple, fast analysis Small sample volume Low power consumption Efficient control and manipulation	Difficulty of fabricating, packaging interfacing Difficulty of multiplexing and reuse	[21, 112–116]
LOAD	Sole controlled by centrifugal forces Highly independent and parallelized reactions	Centrifugal forces Capillary forces Euler forces Coriolis forces	Easy to control Easy and economical to design and fabricate Multiple, independent and parallelized reactions	Limited available materials Hard for small volumes Difficulty of reuse	[39, 53, 60, 73–75]
μPADs	Sole controlled by capillary forces Hydrophilic and porous nature Fold ability Low-cost Ease of use	Capillary forces	Biocompatibility with various substrates Lightweight, flexibility, fold ability, ease of use and availability Low-cost	Imprecise and solo control of flow Lack of detection speed and sensitivity Difficulty of multiplexing and reuse	[133–135]
LFA	Sole controlled by capillary forces Pre-stored chemicals Naked eye read out by color change Low-cost	Capillary forces	Fast Low-cost Easy to operate Equipment-independent Easy to fabricate and miniaturize	Solo assay Hard for quantitative detection Difficulty of multiplexing and reuse Low throughput	[85, 137, 138]

*LOCC* lab on a cartridge chip, *LOAD* lab on a disc, *µPAD* microfluidic paper-based analytical device, *LFA* lateral flow assay

## Digital nucleic acid assay

A digital assay partitions a sample into many microreactors, and each contains a discrete number of target molecules [139, 140]. A digital assay offers significant advantages for performing absolutely quantitative assays by simultaneously and individually conducting thousands of parallel biochemical experiments in micrometer-sized compartments instead of the continuous phase. Reactions in compartments can reduce sample volumes, improve reaction efficiency, and easily integrate with other analytic techniques without the need for networks of channels, pumps, valves, and compact design compared to traditional microfluidics [141–147]. The following two approaches are used for the digital assay to accomplish uniform and precise compartmentalization of solutions, including reagents and samples, such as cells, nucleic acids, and other particles or molecules: (1) droplet emulsions exploiting the interfacial instability of liquids; and (2) array separation through the geometric constraints of the device. In the former method, droplets containing reagents and samples in microchannels can be generated by passive methods, such as co-flow, cross-flow, flow-focusing, step emulsification, microchannel emulsification, and membrane emulsification through viscous shear forces and variations of channel confinement [143, 145, 146, 148, 149], or by active methods with the aid of additional energy input through electrical, magnetic, thermal, and mechanical controls [150, 151]. In the latter method, better uniformity of liquid volume in microfluidic chambers is partitioned by restricting to spatial structures of the same size, for example, microwell and surface arrays [152–154]. Notably, droplets are the mainstream partitions and can also be generated and manipulated on an array of electrodes, which is based on digital microfluidics (DMF). Electrowetting on dielectric is one of the most intensively studied theories in DMF because electrowetting on dielectric is able to control over fluid shape and flow through asymmetric electrical signals on different sides, making precise manipulations of single droplets possible [141, 144]. Basic manipulations of droplets in DMF include sorting, splitting, and merging [151, 155, 156], which can be applied to various analytic fields, especially in molecule detection [157–159].

**Fig. 7:**
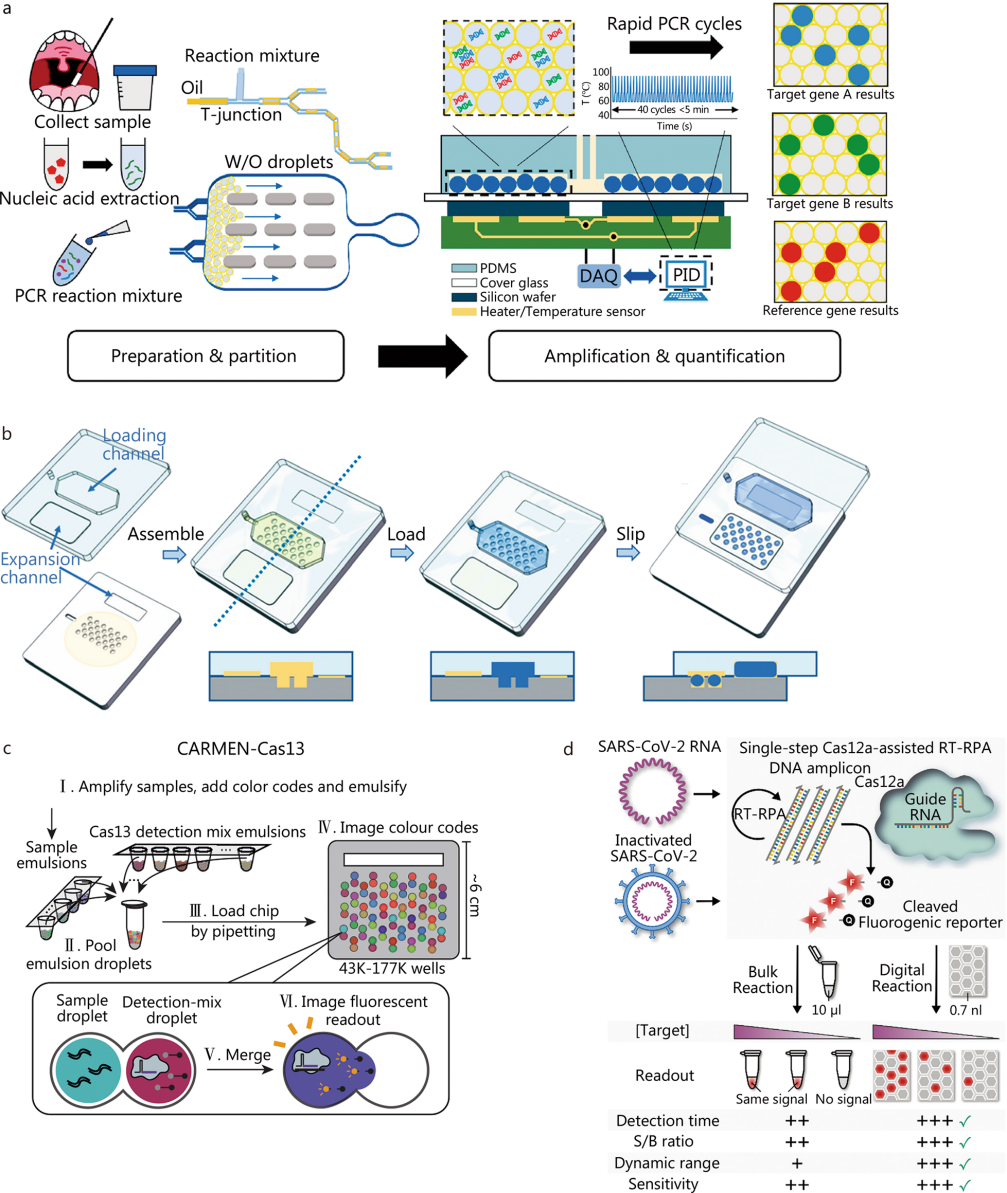
Typic digital nucleic acid assay platforms. **a** Workflow of rapid digital PCR method consists of four key steps: sample preparation, reaction mixture partition, amplification process, and targets quantification (adapted from [164]). **b** Schematic drawings demonstrate the droplet assay SlipChip for slip formation of droplets at high density (adapted from [71]). **c** Schematic of CARMEN-Cas13 workflow (adapted from [158]). **d** Overview of digitization-enhanced CRISPR/Cas-assisted one-pot virus detection (adapted from [169]). W/O water in oil, PDMS polydimethylsiloxane, PCR polymerase chain reaction, DAQ data acquisition, PID proportional integral derivative, CARMEN combinatorial arrayed reactions for multiplexed evaluation of nucleic acids, SARS-CoV-2 severe acute respiratory syndrome coronavirus 2, RT-RPA reverse-transcription recombinase polymerase amplification, S/B signal to background

The digital nucleic acid assay is third-generation technology of molecular diagnostics after conventional PCR and quantitative real-time PCR (qPCR), parallel to high-throughput sequencing and liquid biopsies. The digital nucleic acid has developed quickly in the field of molecular diagnostics to target infectious pathogens in the last two decades [160–162]. The absolute quantification of digital nucleic acid assay begins with packaging samples and reagents into divided compartments to ensure that every target sequence has the same probability to enter every discrete partition. Theoretically, every partition can be assigned a few target sequences or none as an independent micro-reaction system. Through the many kinds of sensing mechanisms discussed above, compartments with the target sequences of microorganisms producing signals above a particular threshold can be visualized by the unaided eye or machines and labeled as positive, while the other compartments producing signals below the threshold are labeled as negative, which makes the signal of every partition Boolean. Therefore, by calculating the number of compartments generated and the positive rate after the reaction, the original copies of the tested samples can be reconciled through the Poisson distribution formula without a standard curve, as is necessary for conventional quantitative detection, like qPCR [163]. Compared with traditional molecular diagnostic technology, the digital nucleic acid assay is much more automatic and integrated with higher analysis velocity and sensitivity, fewer reagents, and lower possibility of pollution, while also easier to design and fabricate. For these reasons, the application of digital assay, especially droplet-based method in molecular diagnostics combining amplification and signal read-out techniques, is well-studied during the crucial outbreak of SARS-CoV-2. For example, Yin et al. [164] combined droplet digital and rapid PCR techniques to detect ORF1ab, N, and RNase P genes in SARS-CoV-2 in a microfluidic chip. Notably, the system can identify a positive signal within 115 s, which is more rapid than conventional PCR, suggesting its efficiency for point-of-care detection (Fig. 7a). Dong et al. [165], Suo et al. [157], Chen et al. [166], and Alteri et al. [167] also applied droplet digital PCR (ddPCR) in microfluidic systems to detect SARS-CoV-2 and achieved impressive research results. To further improve detection speed, Shen et al. [168] realized chip imaging based on ddPCR in just 15 s without applying stitching technology for images, which speeds up the lab-to-application process ddPCR technology. Not only thermal amplification technologies, like PCR, but also isothermal amplification techniques are applied for simplified reaction conditions and rapid response time. Lyu et al. [71] designed a droplet assay SlipChip that is capable of producing droplets of various sizes at high density with a single slipping step and quantifying SARS-CoV-2 nucleic acids via digital LAMP (Fig. 7b). As a rapidly growing technology, CRISPR can also play an important role in the digital nucleic acid assay for its convenient colorimetric visualization without additional nucleic acid dyes. Combinatorial arrayed reactions for multiplexed evaluation of nucleic acids were developed by Ackerman et al. [158] to detect 169 human-associated viruses, including SARS-CoV-2, in droplets containing CRISPR-Cas13-based nucleic acid detection reagents in a microwell assay (Fig. 7c). Moreover, isothermal amplification and CRISPR technologies can be utilized in a system to integrate the advantages of both. Park et al. [169] developed a CRISPR/Cas12a-assisted digital assay in commercial microfluidic chips to detect both extracted and heat-inactivated SARS-CoV-2 based on single-step RT-RPA, which outperforms its bulk counterpart with a shorter detection time, higher signal-to-background ratio, wider dynamic range, and better sensitivity (Fig. 7d). Some descriptions of these examples are shown in Table 3.

**Table 3:** Applications of the digital nucleic acid assay in detecting SARS-CoV-2

Compartmentalization methods	Analyte	Amplification methods	Performance	Detecting methods	References
Crossflow/T-junction	SARS-CoV-2 *ORF1ab*, *N* and *RNase P* genes	RT-qPCR	5 copies/test in 5 min	Fluorescent probes	[164]
Flow-focusing by QX200^™^ Droplet Digital^™^ PCR System, Bio-Rad (Pleasanton, California, U.S.)	SARS-CoV-2 *ORF1ab*, *N* and *E* genes	RT-qPCR	2 copies/reaction in 4 h	TaqMan Hydrolysis probe and EvaGreen	[165]
Flow-focusing by QX200^™^ Droplet Digital^™^ PCR System	SARS-CoV-2 *ORF1ab* and *N* genes	RT-qPCR	1.4 copies/reaction	TaqMan PCR reaction mixture	[157]
By head-flattened pipette tips	SARA-CoV-2 *ORF1ab* and *N* genes	RT-qPCR	3.8 copies/reaction in 1.5 h	Thermo Scientific TaqMan 2019-nCoV Assay Kit v1	[166]
Flow-focusing by QX200^™^ Droplet Digital^™^ PCR System	SARS-CoV-2 *RdRp* and *RNase P* genes	RT-PCR	2.9 copies /reaction	GeneFinder^™^ COVID-19 Plus RealAmp Kit, ELITech and Allplex^™^ 2019-nCoV Assay, Seegene	[167]
SlipChip	SARS-CoV-2 NA extracted from the COVID-19 pseudo virus	LAMP	344 copies/ml	LAMP fluorescent dye	[71]
Microwell arrays	Synthetic SARS-CoV-2 targets	CRISPR-Cas13-based SHERLOCK technology	10 copies/ml	Single-stranded DNA fluorogenic reporters	[158]
By QuantStudio chips (ThermoFisher) with 0.7 nl digital reaction wells	SARS-CoV-2 N gene	CRISPR/Cas12a based RT-RPA	1 GE/µl of SARS-CoV-2 RNA and 20 GE/µl of heat-inactivated SARS-CoV-2, qualitative detection in 15 min and quantitative detection in 30 min	Single-stranded DNA fluorogenic reporters	[169]

*RT-qPCR* reverse-transcription quantitative polymerase chain reaction, *LAMP* loop-mediated isothermal amplification, *RT-PCR* reverse-transcription polymerase chain reaction, *CRISPR* clustered regularly interspaced short palindromic repeats, *SHERLOCK* specific high sensitivity enzymatic reporter unlocking, *RT-RPA* reverse-transcription recombinase polymerase amplification, *GE* genome equivalent, *COVID-19* corona virus disease 2019, *SARS-CoV-2* severe acute respiratory syndrome coronavirus 2

The digital nucleic acid assay is developing at high speed in infectious pathogen diagnosis, although some challenges deserve better solutions. First, the generation of partitions, especially droplets, is supposed to be rapid, stable, and uniform, which calls for an efficient and easy-producing method. Methods that depend on complex external pumps and tubes to compartmentalize are doomed to be replaced by convenient methods. Second, adding some surfactants is necessary to stabilize droplets in microfluidic devices, causing additional business costs. Therefore, a less expensive stabilizer or method is required to ensure stabilization in droplet reactions. Third, the measurement of original copies is based on signal read-out technologies, which involve algorithms to identify positive compartments. Program optimization and algorithm innovation are essential processes to achieve fast and accurate results. Our team created a novel Monte Carlo-based statistic modeling for absolute quantification of pathogenic nucleic acids via digital LAMP, the results of which agree with the proposed mathematical model [170]. Lastly, digital assay can cooperate with DMF to perform individual and parallel reactions, leaving a huge space for development. Overall, we are expecting integrated and automatic digital nucleic acid assays applied in infectious pathogen diagnosis to conduct sample-to-result testing and POCT.

## Commercial microfluidic POCT devices

Microfluidic POCT devices exhibit many advantages in in vitro molecular diagnostics, especially in developing areas. Compared with laboratory testing, the operations of microfluidic POCT devices are integrated into a single microfluidic chip, cartridge, and tube, from sample purification-to-nucleic acid amplification and pathogen measurement, while results are easy and rapid to achieve at a comparatively low financial cost [96, 171, 172]. More and more interest has been drawn to microfluidic POCT devices from worldwide manufacturers because of the automatic tests and limited required reagents [19, 173]. Therefore, microfluidic POCT devices show a bright future to molecular diagnostics in urgent or daily situations and deserve further study. Herein we present some typical and current commercial microfluidic POCT devices for molecular diagnostics to show the current state of development.

Biological manufacturers have developed commercial devices to be applied in various fields, such as food security, agricultural product testing, medical diagnosis, animal industry, and environmental testing*.* Among these fields, medical diagnosis, especially molecular diagnostics, is of the greatest relevance to mankind, making the application much more popular. Using SARS-CoV-2 as an example and since the COVID-19 outbreak, microfluidic devices or newly-designed chips targeting the virus have been launched, such as FilmArray^®^ Biofire^®^ [Biofire (Salt Lake City, UT, U.S.)] [174], GenPlex^®^ [BOHUI (Beijing, China)] [175], Vivalytic [BOSCH (Waiblingen, BW, German); Randox (Antrim, N.IRE, UK)] [176], RTisochip^™^-A (CapitalBio) [177], RTisochip^™^-W (CapitalBio) [178, 179], DxLab-2A (CapitalBio) [180], Cue^™^ [Cue health (San Diego, CA, U.S.)] [181], Simplexa^™^ [Focus Diagnostics (Cypress, CA, U.S.)] [182], QuanPLEX [IntelliBio (Qingdao, SD, China)] [183], a microchip based real-time PCR analyzer AriaDNA [Lumex Instruments (Mission, BC, Canada)] [184], Novodiag^®^ [Mobidiag (Espoo, Finland)] [185], Cobas^®^ Liat^®^ [Roche (Indianapolis, IN, U.S.)] [186], iGeneTec MA3000 [Superchip technology (Shanghai, China)] [187], BINAS [Tsinghua University (Beijing, China)]) [188], Visby Medical^™^ [Visby Medical (San Jose, CA, U.S.)] [189] and WizDx^™^ F-150 Real-time PCR Systems [Wizbiosolutions (Seongnam-si, Republic of Korea)] [190]. Most of these devices also have focused on other respiratory viruses, such as influenza, before the epidemic. Sexually transmitted diseases are increasingly gaining attention from the public, leading to the creation of associated equipment. For example, an IO single module system [Binx health (Boston, MA, U.S.)] [191, 192] can detect *Chlamydia trachomatis* that may cause sexually transmitted diseases in just 30 min and Visby Medical^™^ can detect three kinds of sexual pathogens at the same speed, while GenPlex^®^ and Vivalytic can also test for vaginal pathogens, such as human papilloma virus. As an immunologic challenge in human medicine, the detection of HIV in POCT devices is meaningful, so Abbott launched Alere^™^ Q [Abbott (Des Plaines, IL, U.S.)] [193] and matched HIV-1/2 detection chips. The test can be completed in 52 min, requiring only 25 µl of peripheral blood or plasma. Biocartis focus on tumors and launched Idylla^™^ [Biocartis (Mechelen, Belgium)] to target clinically-significant test sites of genes, such as *BRAF*, *KRAS*, *NRAS*, and *EGFR* [194]. Because of the similarity of molecular diagnostic methodologies, many microfluidic devices perform multiplex detection through concurrent testings and even more testing items may be added by product upgrades. For example, iChip-400 [195] and Onestart-1000 [196] from Baicare (Beijing, China), BD MAX^™^ [197] from BD (Sparks, MD, U.S.), FilmArray^®^ Biofire^®^ from Biofire, GeneXpert^®^ Infinity Systems from Cepheid (Sunnyvale, CA, U.S.) [198], Unyvero A50 [199] from Curetis (West Boylston, MA, U.S.), Revogene^®^ [200] from GenePOC (Quebec, PQ, Canada), ePlex [201] from GenMark (Carlsbad, CA, U.S.), AriaDNA from Lumex Instruments, Novodiag^®^ from Mobidiag, Verigene^®^ [202] from Nanosphere (Beverly, MA, U.S.), and Visby Medical^™^ from Visby Medical. These devices greatly increase the efficiency of the screening and diagnosis. More details of all the aforementioned devices are shown in Table 4.

**Table 4:** Comparison of microfluidic POCT devices for molecular diagnostics

Approval time	Devices	Manufacturers	Regulator	Detection technology	Targets	LOD	Detection time	References
2007.08	Unyvero A50	Curetis	CE	Multiplex PCR	Hospitalized pneumonia, blood culture, intra-abdominal infection, urinary tract infection, implant & tissue infection	–	< 5 h	[199]
2007.09	Verigene^®^	Nanosphere	FDA	RT-PCR	*C. diff*, enteric pathogens, respiratory pathogens, Gram bacteria	–	2–2.5 h	[202]
2010.05	Simplexa^™^	Focus Diagnostics	FDA	RT-PCR	SARS-CoV-2, influenza A/B & RSV, HSV 1 & 2, influenza A, H1N1	242–500 copies/ml	< 80 min	[182]
2011.08	Cobas^®^ Liat^®^	Roche	FDA	Multiplex real-time RT-PCR	SARS-CoV-2, influenza A/B, RSV, Cdiff and strep A	10^–3^–10^–1^ TCID_50_/ml	< 20 min	[186]
2012.01	BD MAX^™^	BD	FDA	RT-PCR	GBS, MRSA, *C. diff*, SA, vaginal pathogens, enteric bacterial and parasite, CT/GC/TV	–	45–90 min	[197]
2012.11	GeneXpert^®^ Infinity Systems	Cepheid	FDA	RT-PCR	GBS, MRSA, gastrointestinal pathogens, MTB, TV, respiratory viruses and so on	–	–	[198]
2013.02	FilmArray^®^ Biofire^®^	Biofire	FDA	Nested Multiplex PCR	Respiratory, blood, gastrointestinal and meningitis infection and so on	1000 TCID_50_/ml	45–60 min	[174]
2014	Idylla^™^	Biocartis	CE	RT-PCR	*BRAF*, *KRAS*, *NRAS*, *EGFR* genes	–	90–180 min	[194]
2015	Alere^™^ Q	Abbott	CE	RT-PCR	HIV	–	< 52 min	[193]
2016.02	IO single module system	Binx health	CE	PCR	CT, NG and so on	–	< 30 min	[191, 192]
2017.05	Revogene^®^	GenePOC	FDA	RT-PCR	*C. diff*, GBS, GAS and CRE	–	< 70 min	[200]
2017.06	ePlex	GenMark	FDA	RT-PCR	Bloodstream infections and respiratory pathogens	–	< 90 min	[201]
2017.07	RTisochip^™^-A	CapitalBio Technology	NMPA	Isothermal amplification	19 respiratory viruses and 8 pathogenic bacteria	10–10^3^ copies/run	< 50 min	[177]
2018.09	iChip-400	Baicare	NMPA	LAMP	16 pathogenic bacteria	–	< 1 h	[195]
2018.11	GenPlex^®^	BOHUI	NMPA	Multiplex PCR	24 HPV and 18 respiratory viruses	–	–	[175]
2019.07	RTisochip^™^-W	CapitalBio Technology	NMPA	NASBA	19 respiratory viruses and 8 pathogenic bacteria	50 copies/run	20–50 min	[178, 179]
2020.01	WizDx^™^ F-150 Real-time PCR System	Wizbiosolutions	CE	Ultra-fast RT-PCR	SARS-CoV-2	20 copies/run	< 40 min	[190]
2021.01	Onestart-1000	Baicare	NMPA	RT-PCR	60 pathogenic bacteria	10^3^ copies/ml	< 1.5 h	[196]
2021.02	DxLab-2A	CapitalBio Technology	NMPA	Nested PCR	SARS-CoV-2	500 copies/ml	< 45 min	[180]
2021.03	Cue^™^	Cue health	FDA	Isothermal amplification	SARS-CoV-2	20 copies/test	20 min	[181]
2021.06	iGeneTec MA3000	Superchip technology	NMPA	Isothermal amplification	SARS-CoV-2	500 copies/ml	< 45 min	[187]
2021.08	Visby Medical^™^	Visby Medical	FDA	PCR	SARS-CoV-2, chlamydia, NG, TV	–	< 30 min	[189]
–	Vivalytic	BOSCH, Randox	–	End-point PCR	Respiratory and sexually transmitted infections viruses	–	30–150 min	[176]
–	QuanPLEX	IntelliBio	–	qPCR	7 respiratory viruses	–	–	[183]
–	AriaDNA	Lumex Instruments	–	RT-PCR	SARS-CoV-2, african swine fever, cattle pathogens, fish pathogens and avian pathogens	9000 copies/ml	< 50 min	[184]
–	Novodiag^®^	Mobidiag	–	RT-PCR	SARS-CoV-2, *C. diff*, enteric pathogens and so on	–	< 1 h	[185]
–	BINAS	Tsinghua University	–	Nested isothermal amplification	SARS-CoV-2	< 400 copies/ml	< 30 min	[188]

"–" means the information is not officially available from public data or is too complicated to be noted; *LOD* limits of detection, *NMPA* national medical products administration; *CE* conformite europeenne, *FDA* U.S. Food & Drug Administration, *C. diff Clostridium difficile, CRE Carbapenem-resistant Enterobacteriaceae, GAS Group A streptococcus; GBS Group B* streptococcus, *HPV* human papilloma virus*, HSV* herpes simplex virus*, MRSA* methicillin-resistant *Staphylococcus aureus*, *MTB Mycobacterium tuberculosis*, *NG Neisseria gonorrhoeae, RSV respiratory syncytial virus, SA Staphylococcus aureus, TV Trichomonas vaginalis, SARS-CoV-2* severe acute respiratory syndrome coronavirus 2, *PCR* polymerase chain reaction, *qPCR* quantitative real-time polymerase chain reaction, *LAMP* loop-mediated isothermal amplification, *RT-PCR* reverse-transcription polymerase chain reaction, *NASBA* nucleic acid sequence-based amplification, *TCID*_*50*_ 50% tissue culture infective dose

Commercial microfluidic POCT devices are attempting to keep up with the modern medical tests conducted at hospitals and laboratories on test sensitivity and specificity. Commercial microfluidic POCT devices are showing exclusive advantages, especially in detecting diversity and degree of integration. In an epidemic, commercial microfluidic devices are of great importance in diagnosis because of efficient technologies, optimized reaction conditions, and convenience without the need for a large-scale laboratory or testing center. Because commercial microfluidic POCT devices were developed during a disease outbreak in a short time by transplanting conventional steps, there is still much room for development with respect to the level of efficiency, automation, integration, sensitivity, specificity, portability, and affordability, thus making the industry in its early stage of application. We are expecting qualified techniques combined with elaborately designed and fabricated chips to get sample-to-result instruments.

## Conclusions

Infectious diseases are posing problems for public medical systems and attracted much attention from the public and scientists. Microfluidics is one of the best technologies to conduct molecular diagnostics for infectious diseases and has made great achievements, especially during the outbreak of COVID-19. In this review we presented the applications of microfluidics-based strategies for infectious disease detection. In the first part, we systematically discussed the common processes of molecular testing based on microfluidics, including sample preprocessing (silicon-, magnetic-, and porous materials-based strategies), nucleic acid amplification (PCR, isothermal amplification, and CRISPR-based amplification-free methods), and signal reading-out (electrochemical, fluorescence, colorimetric, chemiluminescence, surface plasmon resonance-based, and magnetic-based biosensors). Next, various microfluidic platforms, including LOCC, LOAD, μPADs, and LFA, were compared to highlight the features, advantages, and disadvantages. We further discussed and emphasized the novel applications of the digital nucleic acid assay for absolute quantification. Subsequently, we investigated 27 commercial microfluidics-based POCT devices for molecular diagnostics from a decade ago and displayed the targeting objectives and performances.

There is still ample room for the development of microfluidics to deal with the severe, ongoing pandemic. More significantly, new infectious diseases may emerge in the near future. The traditional technologies are mature and optimized, but require multiple steps and frequent transfers of samples between platforms. These sophisticated processes lead to unnecessary pollution and complicated manual operations. Thus, the trend of fully integrated microfluidics is unstoppable, which combines sampling, sensing, and signaling modules. In the sampling module, a large quantity of molecules is expected to be extracted from the limit sample, therefore providing efficient cleavage enzymes, nucleic acid transport carriers, and cleaning agents. In the sensing module, the false-negative results caused by low-sensitive detection usually lead to misdiagnosis and create a burden in the public medical system. Prevention of pandemics calls for high-throughput testing that can precisely detect very few nucleic acids. In practical applications, the diagnostic requirements are so diverse that multiplex diagnostics are more suitable for future tests with expended testing items. In the signaling module, great efforts have been made to accurately identify signals transformed from amplified molecules by algorithms incorporating artificial intelligence, avoiding errors and limitations of manual judgments. There are also some novel strategies still in their early stages; for example, target molecules can be directly tested from sample solutions omitting the pretreatment process. Therefore, nucleic acids must be specifically distinguished between cluttered background molecules, which is challenging. In addition, detecting nucleic acids bypassing sensing modules demands more sensitive testing methods, such as CRISPR, which can respond significantly to individual molecules.

Moreover, the industrialization of microfluidics is still in the start-up phase, reflected in complex channel design, expensive substrate materials, necessary optimization of reactions, liquid leakage, valve failure, and difficulty in reproduction and recyclability. These issues are the main barriers for large-scale adoption, so further improvement is needed to build a convenient chip-design platform, combined with material science to find less expensive substrate substitutes, enhance functional modularity, and push automation of chip producing*.* Fortunately, in dealing with infectious diseases currently and in the future, microfluidic-based molecular diagnostic strategies are indispensable and receiving more attention from frontier scientists. Microfluidics-based molecular diagnostic strategies will become the mainstream of large-scale detection, utilizing rarely required samples, to conduct diagnosis automatically at a low cost.

## Data Availability

Not applicable.

## Abbreviations

COVID-19: Coronavirus disease 2019
CFU: Colony-forming units
CRISPR: Clustered regularly interspaced short palindromic repeats
ddPCR: Digital droplet polymerase chain reaction
DMF: Digital microfluidics
FTA: Finders technology associates
HCV: Hepatitis C virus
HIV: Human immunodeficiency virus
ITP: Isotachophoresis
LAMP: Loop-mediated isothermal amplification
LFA: Lateral flow assay
LOAD: Lab on a disc
LOCC: Lab on a cartridge chip
PCR: Polymerase chain reaction
POCT: Point-of-care testing
qPCR: Quantitative real-time polymerase chain reaction
RPA: Recombinase polymerase amplification
RT-LAMP: Reverse-transcription loop-mediated isothermal amplification
RT-qPCR: Reverse-transcription quantitative polymerase chain reaction
RT-RPA: Reverse-transcription recombinase polymerase amplification
SARS-CoV-2: Severe acute respiratory syndrome coronavirus 2
μPADs: Microfluidic paper-based analytical devices

## References

[1] Stockmaie S, Stroeymeyt N, Shattuck EC, Hawley DM, Meyers LA, Bolnick DI (2021). Infectious diseases and social distancing in nature. Science.

[2] Heesterbeek H, Anderson RM, Andreasen V, Bansal S, De Angelis D, Dye C (2015). Modeling infectious disease dynamics in the complex landscape of global health. Science.

[3] Ali MA, Hu C, Jahan S, Yuan B, Saleh MS, Ju E (2021). Sensing of COVID-19 antibodies in seconds via aerosol jet nanoprinted reduced-graphene-oxide-coated 3D electrodes. Adv Mater.

[4] Kim HS, Abbas N, Shin S (2021). A rapid diagnosis of SARS-CoV-2 using DNA hydrogel formation on microfluidic pores. Biosens Bioelectron.

[5] Rajakaruna SJ, Liu WB, Ding YB, Cao GW (2017). Strategy and technology to prevent hospital-acquired infections: lessons from SARS, Ebola, and MERS in Asia and West Africa. Mil Med Res.

[6] Hospenthal DR, Murray CK (2011). Preface: guidelines for the prevention of infections associated with combat-related injuries: 2011 update. J Trauma.

[7] Atencia J, Beebe DJ (2005). Controlled microfluidic interfaces. Nature.

[8] Sackmann EK, Fulton AL, Beebe DJ (2014). The present and future role of microfluidics in biomedical research. Nature.

[9] Bakas S, Uttamchandani D, Toshiyoshi H, Bauer R (2021). MEMS enabled miniaturized light-sheet microscopy with all optical control. Sci Rep.

[10] Syedmoradi L, Daneshpour M, Alvandipour M, Gomez FA, Hajghassem H, Omidfar K (2017). Point of care testing: the impact of nanotechnology. Biosens Bioelectron.

[11] Jin YH, Zhan QY, Peng ZY, Ren XQ, Yin XT, Cai L (2020). Chemoprophylaxis, diagnosis, treatments, and discharge management of COVID-19: an evidence-based clinical practice guideline (updated version). Mil Med Res.

[12] Guo YR, Cao QD, Hong ZS, Tan YY, Chen SD, Jin HJ (2020). The origin, transmission and clinical therapies on coronavirus disease 2019 (COVID-19) outbreak—an update on the status. Mil Med Res.

[13] Jin YH, Huang Q, Wang YY, Zeng XT, Luo LS, Pan ZY (2020). Perceived infection transmission routes, infection control practices, psychosocial changes, and management of COVID-19 infected healthcare workers in a tertiary acute care hospital in Wuhan: a cross-sectional survey. Mil Med Res.

[14] Telenti A, Arvin A, Corey L, Corti D, Diamond MS, García-Sastre A (2021). After the pandemic: perspectives on the future trajectory of COVID-19. Nature.

[15] Wang Y, Wang JY, Schnieke A, Fischer K (2021). Advances in single-cell sequencing: insights from organ transplantation. Mil Med Res.

[16] Liu W, Yue F, Lee LP (2021). Integrated point-of-care molecular diagnostic devices for infectious diseases. Acc Chem Res.

[17] Bhalla N, Pan Y, Yang Z, Payam AF (2020). Opportunities and challenges for biosensors and nanoscale analytical tools for pandemics: COVID-19. ACS Nano.

[18] Pang NYL, Pang ASR, Chow VT, Wang DY (2021). Understanding neutralising antibodies against SARS-CoV-2 and their implications in clinical practice. Mil Med Res.

[19] Tarim EA, Karakuzu B, Oksuz C, Sarigil O, Kizilkaya M, Al-Ruweidi MKA (2021). Microfluidic-based virus detection methods for respiratory diseases. Emergent Mater.

[20] Yin J, Suo Y, Zou Z, Sun J, Zhang S, Wang B (2019). Integrated microfluidic systems with sample preparation and nucleic acid amplification. Lab Chip.

[21] Sun F, Ganguli A, Nguyen J, Brisbin R, Shanmugam K, Hirschberg DL (2020). Smartphone-based multiplex 30-min nucleic acid test of live virus from nasal swab extract. Lab Chip.

[22] Emaus MN, Varona M, Eitzmann DR, Hsieh SA, Zeger VR, Anderson JL (2020). Nucleic acid extraction: fundamentals of sample preparation methodologies, current advancements, and future endeavors. TrAC Trends Anal Chem.

[23] Brassard D, Geissler M, Descarreaux M, Tremblay D, Daoud J, Clime L (2019). Extraction of nucleic acids from blood: unveiling the potential of active pneumatic pumping in centrifugal microfluidics for integration and automation of sample preparation processes. Lab Chip.

[24] Urbaniak J, Janowski D, Jacewski B (2019). Isolation of nucleic acids using silicon dioxide powder as a tool for environmental monitoring. Environ Monit Assess.

[25] Soares RR, Neumann F, Caneira CR, Madaboosi N, Ciftci S, Hernández-Neuta I (2019). Silica bead-based microfluidic device with integrated photodiodes for the rapid capture and detection of rolling circle amplification products in the femtomolar range. Biosens Bioelectron.

[26] Liu J, Zhao J, Petrochenko P, Zheng J, Hewlett I (2016). Sensitive detection of influenza viruses with Europium nanoparticles on an epoxy silica sol-gel functionalized polycarbonate–polydimethylsiloxane hybrid microchip. Biosens Bioelectron.

[27] Kinahan DJ, Mangwanya F, Garvey R, Chung DW, Lipinski A, Julius LA, et al. Automation of silica bead-based nucleic acid extraction on a centrifugal lab-on-a-disc platform. In: 27th Micromechanics and microsystems Europe (MME) workshop. 2016; p. 012013.

[28] Kang JH, Kim YT, Lee K, Kim HM, Lee KG, Ahn J (2020). An electrophoretic DNA extraction device using a nanofilter for molecular diagnosis of pathogens. Nanoscale.

[29] Lee K, Kang JH, Kim HM, Ahn J, Lim H, Lee J (2020). Direct electrophoretic microRNA preparation from clinical samples using nanofilter membrane. Nano Converg.

[30] Wang X, Yan C, Wang X, Zhao X, Shi C, Ma C (2020). Integrated silica membrane-based nucleic acid purification, amplification, and visualization platform for low-cost, rapid detection of foodborne pathogens. Anal Bioanal Chem.

[31] Powell L, Wiederkehr RS, Damascus P, Fauvart M, Buja F, Stakenborg T (2018). Rapid and sensitive detection of viral nucleic acids using silicon microchips. Analyst.

[32] Chen S, Chen X, Du J, Zhang Y, Yang H. In-flow extraction of RNA in extracellular vesicles using a silicon-based microfluidic device. In: 2021 IEEE 34th International conference on micro electro mechanical systems (MEMS). 2021; p. 1015–8.

[33] Sharif S, Wang Y, Ye Z, Wang Z, Qiu Q, Ying S (2019). A novel impedimetric sensor for detecting LAMP amplicons of pathogenic DNA based on magnetic separation. Sens Actuators B.

[34] Sun L, Siddique KM, Wang L, Li S (2021). Mixing characteristics of a bubble mixing microfluidic chip for genomic DNA extraction based on magnetophoresis: CFD simulation and experiment. Electrophoresis.

[35] Bai Y, Cui Y, Paoli GC, Shi C, Wang D, Zhou M (2016). Synthesis of amino-rich silica-coated magnetic nanoparticles for the efficient capture of DNA for PCR. Colloids Surf B.

[36] Li P, Li M, Zhang F, Wu M, Jiang X, Ye B (2021). High-efficient nucleic acid separation from animal tissue samples via surface modified magnetic nanoparticles. Sep Purif Technol.

[37] Mosley O, Melling L, Tarn MD, Kemp C, Esfahani MM, Pamme N (2016). Sample introduction interface for on-chip nucleic acid-based analysis of *Helicobacter pylori* from stool samples. Lab Chip.

[38] Rodriguez-Mateos P, Ngamsom B, Walter C, Dyer CE, Gitaka J, Iles A (2021). A lab-on-a-chip platform for integrated extraction and detection of SARS-CoV-2 RNA in resource-limited settings. Anal Chim Acta.

[39] Dignan LM, Woolf MS, Tomley CJ, Nauman AQ, Landers JP (2021). Multiplexed centrifugal microfluidic system for dynamic solid-phase purification of polynucleic acids direct from buccal swabs. Anal Chem.

[40] Byrnes SA, Bishop JD, Lafleur L, Buser JR, Lutz B, Yager P (2015). One-step purification and concentration of DNA in porous membranes for point-of-care applications. Lab Chip.

[41] Zhang L, Zhang Y, Wang C, Feng Q, Fan F, Zhang G (2014). Integrated microcapillary for sample-to-answer nucleic acid pretreatment, amplification, and detection. Anal Chem.

[42] Lu W, Wang J, Wu Q, Sun J, Chen Y, Zhang L (2016). High-throughput sample-to-answer detection of DNA/RNA in crude samples within functionalized micro-pipette tips. Biosens Bioelectron.

[43] Shen KM, Sabbavarapu NM, Fu CY, Jan JT, Wang JR, Hung SC (2019). An integrated microfluidic system for rapid detection and multiple subtyping of influenza A viruses by using glycan-coated magnetic beads and RT-PCR. Lab Chip.

[44] Chen P, Chen C, Liu Y, Du W, Feng X, Liu BF (2019). Fully integrated nucleic acid pretreatment, amplification, and detection on a paper chip for identifying EGFR mutations in lung cancer cells. Sens Actuators B.

[45] Connelly JT, Rolland JP, Whitesides GM (2015). “Paper machine” for molecular diagnostics. Anal Chem.

[46] Choi JR, Yong KW, Tang R, Gong Y, Wen T, Li F (2017). Advances and challenges of fully integrated paper-based point-of-care nucleic acid testing. TrAC Trends Anal Chem.

[47] Panahi E, Shivas M, Hall-Mendelin S, Kurucz N, Rudd PA, De Araujo R (2020). Utilising a novel surveillance system to enhance field screening activities for the leishmaniases. MethodsX.

[48] Hashimoto M, Bando M, Kido JI, Yokota K, Mita T, Kajimoto K (2019). Nucleic acid purification from dried blood spot on FTA Elute Card provides template for polymerase chain reaction for highly sensitive Plasmodium detection. Parasitol Int.

[49] Chauhan P, Gupta P, Chhikara K, Goyal K, Singh MP (2021). FTA cards for COVID 2019 samples: easy and cost effective innovation!. Virusdisease.

[50] Kadimisetty K, Yin K, Roche AM, Yi Y, Bushman FD, Collman RG (2021). An integrated self-powered 3D printed sample concentrator for highly sensitive molecular detection of HIV in whole blood at the point of care. Analyst.

[51] Gan W, Gu Y, Han J, Li CX, Sun J, Liu P (2017). Chitosan-modified filter paper for nucleic acid extraction and “in situ PCR” on a thermoplastic microchip. Anal Chem.

[52] Zhu X, Zhao J, Hu A, Pan J, Deng G, Hua C (2020). A novel microfluidic device integrated with chitosan-modified capillaries for rapid ZIKV detection. Micromachines (Basel).

[53] Xiong H, Ye X, Li Y, Qi J, Fang X, Kong J (2021). Efficient microfluidic-based air sampling/monitoring platform for detection of aerosol SARS-CoV-2 on-site. Anal Chem.

[54] Parihar A, Ranjan P, Sanghi SK, Srivastava AK, Khan R (2020). Point-of-care biosensor-based diagnosis of COVID-19 holds promise to combat current and future pandemics. ACS Appl Bio Mater.

[55] Arshavsky-Graham S, Segal E (2020). Lab-on-a-chip devices for point-of-care medical diagnostics. Adv Biochem Eng Biotechnol.

[56] Jiang N, Tansukawat ND, Gonzalez-Macia L, Ates HC, Dincer C, Guder F (2021). Low-cost optical assays for point-of-care diagnosis in resource-limited settings. ACS Sens.

[57] Zhu H, Zhang H, Xu Y, Laššáková S, Korabečná M, Neužil P (2020). PCR past, present and future. Biotechniques.

[58] Watanabe R, Asai S, Kakizoe H, Saeki H, Masukawa A, Miyazawa M (2021). Evaluation of the basic assay performance of the GeneSoc(R) rapid PCR testing system for detection of severe acute respiratory syndrome coronavirus 2. PLoS ONE.

[59] Dong X, Liu L, Tu Y, Zhang J, Miao G, Zhang L (2021). Rapid PCR powered by microfluidics: a quick review under the background of COVID-19 pandemic. TrAC Trends Anal Chem.

[60] Ji M, Xia Y, Loo JFC, Li L, Ho HP, He J (2020). Automated multiplex nucleic acid tests for rapid detection of SARS-CoV-2, influenza A and B infection with direct reverse-transcription quantitative PCR (dirRT-qPCR) assay in a centrifugal microfluidic platform. RSC Adv.

[61] Park YM, Park J, Lim SY, Kwon Y, Bae NH, Park JK (2021). Integrated pumpless microfluidic chip for the detection of foodborne pathogens by polymerase chain reaction and electrochemical analysis. Sens Actuators B.

[62] Ma SY, Chiang YC, Hsu CH, Chen JJ, Hsu CC, Chao AC (2019). Peanut detection using droplet microfluidic polymerase chain reaction device. J Sens.

[63] Jung JH, Choi SJ, Park BH, Choi YK, Seo TS (2012). Ultrafast Rotary PCR system for multiple influenza viral RNA detection. Lab Chip.

[64] Saito M, Takahashi K, Kiriyama Y, Espulgar WV, Aso H, Sekiya T (2017). Centrifugation-controlled thermal convection and its application to rapid microfluidic polymerase chain reaction devices. Anal Chem.

[65] Augustine R, Hasan A, Das S, Ahmed R, Mori Y, Notomi T (2020). Loop-mediated isothermal amplification (LAMP): a rapid, sensitive, specific, and cost-effective point-of-care test for coronaviruses in the context of COVID-19 pandemic. Biology (Basel).

[66] Salazar A, Ochoa-Corona FM, Talley JL, Noden BH (2021). Recombinase polymerase amplification (RPA) with lateral flow detection for three Anaplasma species of importance to livestock health. Sci Rep.

[67] Li B, Zou B, Ma X, Wu H, Zhang Y, Zhou G (2021). Research progress in technologies based on isothermal amplification of nucleic acids for detection of SARS-CoV-2. Chin J Virol.

[68] Chen P, Chen C, Su H, Zhou M, Li S, Du W (2021). Integrated and finger-actuated microfluidic chip for point-of-care testing of multiple pathogens. Talanta.

[69] Sayad A, Ibrahim F, Uddin SM, Cho J, Madou M, Thong KL (2018). A microdevice for rapid, monoplex and colorimetric detection of foodborne pathogens using a centrifugal microfluidic platform. Biosens Bioelectron.

[70] Chen C, Liu P, Zhao X, Du W, Feng X, Liu BF (2017). A self-contained microfluidic in-gel loop-mediated isothermal amplification for multiplexed pathogen detection. Sens Actuators B.

[71] Lyu W, Zhang J, Yu Y, Xu L, Shen F (2021). Slip formation of a high-density droplet array for nucleic acid quantification by digital LAMP with a random-access system. Lab Chip.

[72] Tian F, Liu C, Deng J, Han Z, Zhang L, Chen Q (2020). A fully automated centrifugal microfluidic system for sample-to-answer viral nucleic acid testing. Sci China Chem.

[73] de Oliveira KG, Estrela PFN, de Melo MG, Dos Santos CA, de Paula S-L, Duarte GRM (2021). Rapid molecular diagnostics of COVID-19 by RT-LAMP in a centrifugal polystyrene-toner based microdevice with end-point visual detection. Analyst.

[74] Xiong H, Ye X, Li Y, Wang L, Zhang J, Fang X (2020). Rapid differential diagnosis of seven human respiratory coronaviruses based on centrifugal microfluidic nucleic acid assay. Anal Chem.

[75] Soares RRG, Akhtar AS, Pinto IF, Lapins N, Barrett D, Sandh G (2021). Sample-to-answer COVID-19 nucleic acid testing using a low-cost centrifugal microfluidic platform with bead-based signal enhancement and smartphone read-out. Lab Chip.

[76] Xia Y, Liu Z, Yan S, Yin F, Feng X, Liu BF (2016). Identifying multiple bacterial pathogens by loop-mediated isothermal amplification on a rotate & react slipchip. Sens Actuators B.

[77] Hu JQ, Wei XK, Huang RN, Sun XC, Jing JZ, Gao H (2018). Advance in RPA detection technologies of foodborne pathogenic bacteria. Sci Technol Food Ind.

[78] Bender AT, Sullivan BP, Zhang JY, Juergens DC, Lillis L, Boyle DS (2021). HIV detection from human serum with paper-based isotachophoretic RNA extraction and reverse transcription recombinase polymerase amplification. Analyst.

[79] Schulz M, Calabrese S, Hausladen F, Wurm H, Drossart D, Stock K (2020). Point-of-care testing system for digital single cell detection of MRSA directly from nasal swabs. Lab Chip.

[80] Zheng C, Wang K, Zheng W, Cheng Y, Li T, Cao B (2021). Rapid developments in lateral flow immunoassay for nucleic acid detection. Analyst.

[81] Xin L, Zhang L (2020). Recent progress in nucleic acid-microfluidic chips used for detection of foodborne pathogens: a review. Food Sci.

[82] Yang B, Kong J, Fang X (2019). Bandage-like wearable flexible microfluidic recombinase polymerase amplification sensor for the rapid visual detection of nucleic acids. Talanta.

[83] Yin J, Zou Z, Hu Z, Zhang S, Zhang F, Wang B (2020). A "sample-in-multiplex-digital-answer-out" chip for fast detection of pathogens. Lab Chip.

[84] Fan Y, Wang S, Li Q, Hu Y, Song M, Qin F (2021). Rapid detection of Shiga toxin-producing *Escherichia coli* by recombinase polymerase amplification combined with centrifugal compact disc microfluidic chip. Food Sci.

[85] Liu D, Shen H, Zhang Y, Shen D, Zhu M, Song Y (2021). A microfluidic-integrated lateral flow recombinase polymerase amplification (MI-IF-RPA) assay for rapid COVID-19 detection. Lab Chip.

[86] Kong M, Li Z, Wu J, Hu J, Sheng Y, Wu D (2019). A wearable microfluidic device for rapid detection of HIV-1 DNA using recombinase polymerase amplification. Talanta.

[87] Kellner MJ, Koob JG, Gootenberg JS, Abudayyeh OO, Zhang F (2019). SHERLOCK: nucleic acid detection with CRISPR nucleases. Nat Protoc.

[88] Broughton JP, Deng X, Yu G, Fasching CL, Servellita V, Singh J (2020). CRISPR-Cas12-based detection of SARS-CoV-2. Nat Biotechnol.

[89] Li Y, Li T, Liu BF, Hu R, Zhu J, He T (2020). CRISPR-Cas12a trans-cleaves DNA G-quadruplexes. Chem Commun (Camb).

[90] Li T, Hu R, Xia J, Xu Z, Chen D, Xi J (2021). G-triplex: a new type of CRISPR-Cas12a reporter enabling highly sensitive nucleic acid detection. Biosens Bioelectron.

[91] Qin P, Park M, Alfson KJ, Tamhankar M, Carrion R, Patterson JL (2019). Rapid and fully microfluidic ebola virus detection with CRISPR-Cas13a. ACS Sens.

[92] Chen Y, Mei Y, Zhao X, Jiang X (2020). Reagents-loaded, automated assay that integrates recombinase-aided amplification and Cas12a nucleic acid detection for a point-of-care test. Anal Chem.

[93] Silva FS, Erdogmus E, Shokr A, Kandula H, Thirumalaraju P, Kanakasabapathy MK (2021). SARS-CoV-2 RNA detection by a cellphone-based amplification-free system with CRISPR/CAS-dependent enzymatic (CASCADE) assay. Adv Mater Technol..

[94] Berkenbrock JA, Grecco-Machado R, Achenbach S (2020). Arsenal of microfluidic testing devices may combat COVID-19 pandemic. MRS Bull.

[95] Li C, Zhao C, Bao J, Tang B, Wang Y, Gu B (2020). Laboratory diagnosis of coronavirus disease-2019 (COVID-19). Clin Chim Acta.

[96] Wang C, Liu M, Wang Z, Li S, Deng Y, He N (2021). Point-of-care diagnostics for infectious diseases: from methods to devices. Nano Today.

[97] Tian T, Shu B, Jiang Y, Ye M, Liu L, Guo Z (2021). An ultralocalized Cas13a assay enables universal and nucleic acid amplification-free single-molecule RNA diagnostics. ACS Nano.

[98] Gao Z, Ducos P, Ye H, Zauberman J, Sriram A, Yang X (2020). Graphene transistor arrays functionalized with genetically engineered antibody fragments for Lyme disease diagnosis. 2D Materials.

[99] Wu J, Wang X, Wang Q, Lou Z, Li S, Zhu Y (2019). Nanomaterials with enzyme-like characteristics (nanozymes): next-generation artificial enzymes (II). Chem Soc Rev.

[100] Choi Y, Hwang JH, Lee SY (2018). Recent trends in nanomaterials-based colorimetric detection of pathogenic bacteria and viruses. Small Methods.

[101] Sriram G, Bhat MP, Patil P, Uthappa UT, Jung HY, Altalhi T (2017). Paper-based microfluidic analytical devices for colorimetric detection of toxic ions: a review. TrAC Trends Anal Chem.

[102] Aldewachi H, Chalati T, Woodroofe MN, Bricklebank N, Sharrack B, Gardiner P (2018). Gold nanoparticle-based colorimetric biosensors. Nanoscale.

[103] Oh SJ, Park BH, Choi G, Seo JH, Jung JH, Choi JS (2016). Fully automated and colorimetric foodborne pathogen detection on an integrated centrifugal microfluidic device. Lab Chip.

[104] Liong M, Hoang AN, Chung J, Gural N, Ford CB, Min C (2013). Magnetic barcode assay for genetic detection of pathogens. Nat Commun.

[105] Murzin D, Mapps DJ, Levada K, Belyaev V, Omelyanchik A, Panina L (2020). Ultrasensitive magnetic field sensors for biomedical applications. Sensors (Basel).

[106] Sharma PP, Albisetti E, Massetti M, Scolari M, La Torre C, Monticelli M (2017). Integrated platform for detecting pathogenic DNA via magnetic tunneling junction-based biosensors. Sens Actuators B Chem.

[107] Foudeh AM, Didar TF, Veres T, Tabrizian M (2012). Microfluidic designs and techniques using lab-on-a-chip devices for pathogen detection for point-of-care diagnostics. Lab Chip.

[108] Mu HY, Lu YL, Hsiao TH, Huang JH (2020). Microfluidic-based approaches for COVID-19 diagnosis. Biomicrofluidics.

[109] Haeberle S, Zengerle R (2007). Microfluidic platforms for lab-on-a-chip applications. Lab Chip.

[110] Mark D, Haeberle S, Roth G, Stetten FV, Zengerle R (2010). Microfluidic lab-on-a-chip platforms: requirements, characteristics and applications. Chem Soc Rev.

[111] Stone HA, Stroock AD, Ajdari A (2004). Engineering flows in small devices: microfluidics toward a lab-on-a-chip. Annu Rev Fluid Mech.

[112] Sundah NR, Natalia A, Liu Y, Ho NR, Zhao H, Chen Y (2021). Catalytic amplification by transition-state molecular switches for direct and sensitive detection of SARS-CoV-2. Sci Adv.

[113] Kang BH, Lee Y, Yu ES, Na H, Kang M, Huh HJ (2021). Ultrafast and real-time nanoplasmonic on-chip polymerase chain reaction for rapid and quantitative molecular diagnostics. ACS Nano.

[114] Li X, Zhao X, Yang W, Xu F, Chen B, Peng J (2021). Stretch-driven microfluidic chip for nucleic acid detection. Biotechnol Bioeng.

[115] Ramachandran A, Huyke DA, Sharma E, Sahoo MK, Huang C, Banaei N (2020). Electric field-driven microfluidics for rapid CRISPR-based diagnostics and its application to detection of SARS-CoV-2. Proc Natl Acad Sci U S A.

[116] Jadhav SA, Biji P, Panthalingal MK, Krishna CM, Rajkumar S, Joshi DS (2021). Development of integrated microfluidic platform coupled with surface-enhanced Raman spectroscopy for diagnosis of COVID-19. Med Hypotheses.

[117] Madou M, Zoval J, Jia G, Kido H, Kim J, Kim N (2006). Lab on a CD. Annu Rev Biomed Eng.

[118] Strohmeier O, Keller M, Schwemmer F, Zehnle S, Mark D, Stetten FV (2015). Centrifugal microfluidic platforms: advanced unit operations and applications. Chem Soc Rev.

[119] Lee BS, Lee YU, Kim HS, Kim TH, Park J, Lee JG (2011). Fully integrated lab-on-a-disc for simultaneous analysis of biochemistry and immunoassay from whole blood. Lab Chip.

[120] Kim TH, Park J, Kim CJ, Cho YK (2014). Fully integrated lab-on-a-disc for nucleic acid analysis of food-borne pathogens. Anal Chem.

[121] Loo JFC, Kwok HC, Leung CCH, Wu SY, Law ILG, Cheung YK (2017). Sample-to-answer on molecular diagnosis of bacterial infection using integrated lab-on-a-disc. Biosens Bioelectron.

[122] Loo JFC, Leung CCH, Kwok HC, Wu SY, Law ILG, Chin ML (2017). Rapid molecular diagnosis of bacterial infection using integrated lab-on-a-disc. Procedia Technol.

[123] Sunkara V, Kumar S, Sabaté Del Río J, Kim I, Cho YK (2021). Lab-on-a-disc for point-of-care infection diagnostics. Acc Chem Res.

[124] Nguyen HV, Nguyen VD, Nguyen HQ, Chau THT, Lee EY, Seo TS (2019). Nucleic acid diagnostics on the total integrated lab-on-a-disc for point-of-care testing. Biosens Bioelectron.

[125] Müller RH, Clegg DL (1949). Automatic paper chromatography. Anal Chem.

[126] Martinez AW, Phillips ST, Butte MJ, Whitesides GM (2007). Patterned paper as a platform for inexpensive, low-volume, portable bioassays. Angew Chem Int Ed Engl.

[127] Sachdeva S, Davis RW, Saha AK (2021). Microfluidic point-of-care testing: commercial landscape and future directions. Front Bioeng Biotechnol.

[128] Tang RH, Liu LN, Zhang SF, He XC, Li XJ, Xu F (2019). A review on advances in methods for modification of paper supports for use in point-of-care testing. Mikrochim Acta.

[129] Manmana Y, Kubo T, Otsuka K (2021). Recent developments of point-of-care (POC) testing platform for biomolecules. TrAC Trends Anal Chem.

[130] Yetisen AK, Akram MS, Lowe CR (2013). Paper-based microfluidic point-of-care diagnostic devices. Lab Chip.

[131] Liu R, McConnell EM, Li J, Li Y (2020). Advances in functional nucleic acid based paper sensors. J Mater Chem B.

[132] Hu J, Xiao K, Jin B, Zheng X, Ji F, Bai D (2019). Paper-based point-of-care test with xeno nucleic acid probes. Biotechnol Bioeng.

[133] Teengam P, Nisab N, Chuaypen N, Tangkijvanich P, Vilaivan T, Chailapakul O (2021). Fluorescent paper-based DNA sensor using pyrrolidinyl peptide nucleic acids for hepatitis C virus detection. Biosens Bioelectron.

[134] Lu Q, Su T, Shang Z, Jin D, Shu Y, Xu Q (2021). Flexible paper-based Ni-MOF composite/AuNPs/CNTs film electrode for HIV DNA detection. Biosens Bioelectron.

[135] Chowdury MA, Khalid F (2021). Application of microfluidic paper-based analytical device (μPAD) to detect COVID-19 in energy deprived countries. Int J Energy Res.

[136] Kathrada AI, Wei SC, Xu Y, Cheow LF, Chen CH (2020). Microfluidic compartmentalization to identify gene biomarkers of infection. Biomicrofluidics.

[137] Gong Y, Zheng Y, Jin B, You M, Wang J, Li X (2019). A portable and universal upconversion nanoparticle-based lateral flow assay platform for point-of-care testing. Talanta.

[138] Fu X, Cheng Z, Yu J, Choo P, Chen L, Choo J (2016). A SERS-based lateral flow assay biosensor for highly sensitive detection of HIV-1 DNA. Biosens Bioelectron.

[139] Basu AS (2017). Digital assays part I: partitioning statistics and digital PCR. Slas Technol.

[140] Zhang Y, Noji H (2017). Digital bioassays: theory, applications, and perspectives. Anal Chem.

[141] Choi K, Ng AH, Fobel R, Wheeler AR (2012). Digital microfluidics. Annu Rev Anal Chem (Palo Alto Calif).

[142] Guo MT, Rotem A, Heyman JA, Weitz DA (2012). Droplet microfluidics for high-throughput biological assays. Lab Chip.

[143] Huebner A, Sharma S, Srisa-Art M, Hollfelder F, Edel JB, Demello AJ (2008). Microdroplets: a sea of applications?. Lab Chip.

[144] Nelson WC, Kim CJC (2012). Droplet actuation by electrowetting-on-dielectric (EWOD): a review. J Adhes Sci Technol.

[145] Pompano RR, Liu W, Du W, Ismagilov RF (2011). Microfluidics using spatially defined arrays of droplets in one, two, and three dimensions. Annu Rev Anal Chem (Palo Alto Calif).

[146] Teh SY, Lin R, Hung LH, Lee AP (2008). Droplet microfluidics. Lab Chip.

[147] Theberge AB, Courtois F, Schaerli Y, Fischlechner M, Abell C, Hollfelder F (2010). Microdroplets in microfluidics: an evolving platform for discoveries in chemistry and biology. Angew Chem Int Ed Engl.

[148] Zhu P, Wang L (2016). Passive and active droplet generation with microfluidics: a review. Lab Chip.

[149] Seemann R, Brinkmann M, Pfohl T, Herminghaus S (2012). Droplet based microfluidics. Rep Prog Phys.

[150] Chong ZZ, Tan SH, Gañán-Calvo AM, Tor SB, Loh NH, Nguyen N-T (2016). Active droplet generation in microfluidics. Lab Chip.

[151] Shang L, Cheng Y, Zhao Y (2017). Emerging droplet microfluidics. Chem Rev.

[152] Witters D, Sun B, Begolo S, Rodriguez-Manzano J, Robles W, Ismagilov RF (2014). Digital biology and chemistry. Lab Chip.

[153] Morrison T, Hurley J, Garcia J, Yoder K, Katz A, Roberts D (2006). Nanoliter high throughput quantitative PCR. Nucleic Acids Res.

[154] Rondelez Y, Tresset G, Tabata KV, Arata H, Fujita H, Takeuchi S (2005). Microfabricated arrays of femtoliter chambers allow single molecule enzymology. Nat Biotechnol.

[155] Jeffries GD, Kuo JS, Chiu DT (2007). Dynamic modulation of chemical concentration in an aqueous droplet. Angew Chem Int Ed Engl.

[156] Jeffries GDM, Kuo JS, Chiu DT (2007). Controlled shrinkage and re-expansion of a single aqueous droplet inside an optical vortex trap. J Phys Chem B.

[157] Suo T, Liu X, Feng J, Guo M, Hu W, Guo D (2020). ddPCR: a more accurate tool for SARS-CoV-2 detection in low viral load specimens. Emerg Microbes Infect.

[158] Ackerman CM, Myhrvold C, Thakku SG, Freije CA, Metsky HC, Yang DK (2020). Massively multiplexed nucleic acid detection with Cas13. Nature.

[159] Pollack MG, Pamula VK, Srinivasan V, Eckhardt AE (2011). Applications of electrowetting-based digital microfluidics in clinical diagnostics. Expert Rev Mol Diagn.

[160] Pekin D, Skhiri Y, Baret JC, Le Corre D, Mazutis L, Salem CB (2011). Quantitative and sensitive detection of rare mutations using droplet-based microfluidics. Lab Chip.

[161] Cao L, Cui X, Hu J, Li Z, Choi JR, Yang Q (2017). Advances in digital polymerase chain reaction (dPCR) and its emerging biomedical applications. Biosens Bioelectron.

[162] Liu H, Lei Y (2021). A critical review: recent advances in “digital” biomolecule detection with single copy sensitivity. Biosens Bioelectron.

[163] Whale AS, Huggett JF, Cowen S, Speirs V, Shaw J, Ellison S (2012). Comparison of microfluidic digital PCR and conventional quantitative PCR for measuring copy number variation. Nucleic Acids Res.

[164] Yin H, Wu Z, Shi N, Qi Y, Jian X, Zhou L (2021). Ultrafast multiplexed detection of SARS-CoV-2 RNA using a rapid droplet digital PCR system. Biosens Bioelectron.

[165] Dong L, Zhou J, Niu C, Wang Q, Pan Y, Sheng S (2021). Highly accurate and sensitive diagnostic detection of SARS-CoV-2 by digital PCR. Talanta.

[166] Chen L, Yadav V, Zhang C, Huo X, Wang C, Senapati S (2021). Elliptical pipette generated large microdroplets for POC visual ddPCR quantification of low viral load. Anal Chem.

[167] Alteri C, Cento V, Antonello M, Colagrossi L, Merli M, Ughi N (2020). Detection and quantification of SARS-CoV-2 by droplet digital PCR in real-time PCR negative nasopharyngeal swabs from suspected COVID-19 patients. PLoS ONE.

[168] Shen J, Zheng J, Li Z, Liu Y, Jing F, Wan X (2021). A rapid nucleic acid concentration measurement system with large field of view for a droplet digital PCR microfluidic chip. Lab Chip.

[169] Park JS, Hsieh K, Chen L, Kaushik A, Trick AY, Wang TH (2021). Digital CRISPR/cas-assisted assay for rapid and sensitive detection of SARS-CoV-2. Adv Sci.

[170] Xia Y, Yan S, Zhang X, Ma P, Du W, Feng X (2017). Monte carlo modeling-based digital loop-mediated isothermal amplification on a spiral chip for absolute quantification of nucleic acids. Anal Chem.

[171] Song Q, Sun X, Dai Z, Gao Y, Gong X, Zhou B (2021). Point-of-care testing detection methods for COVID-19. Lab Chip.

[172] Craw P, Balachandran W (2012). Isothermal nucleic acid amplification technologies for point-of-care diagnostics: a critical review. Lab Chip.

[173] Jung W, Han J, Choi JW, Ahn CH (2015). Point-of-care testing (POCT) diagnostic systems using microfluidic lab-on-a-chip technologies. Microelectron Eng.

[174] The BioFire^®^ FilmArray^®^ System. https://www.biofiredx.com/products/filmarray/. Accessed 24 Aug 2021.

[175] GenPlex^®^. http://bohui-tech.com/index.php?m=content&c=index&a=lists&catid=99. Accessed 24 Aug 2021.

[176] Vivalytic. https://www.randox.com/vivalytic-molecular-point-of-care/, https://www.bosch-vivalytic.com/en/vivalytic-overview/. Accessed 24 Aug 2021.

[177] RTisochip^™^-A. http://en.capitalbiotech.com/en/products-content.html?id=34. Accessed 24 Aug 2021.

[178] RTisochip^™^-W. http://en.capitalbiotech.com/en/products-content.html?id=1665. Accessed 24 Aug 2021.

[179] Xing W, Liu Y, Wang H, Li S, Lin Y, Chen L (2020). A high-throughput, multi-index isothermal amplification platform for rapid detection of 19 types of common respiratory viruses including SARS-CoV-2. Engineering (Beijing).

[180] Xing W, Wang J, Zhao C, Wang H, Bai L, Pan L (2021). A highly automated mobile laboratory for on-site molecular diagnostics in the COVID-19 pandemic. Clin Chem.

[181] Cue's COVID-19 diagnostic test. https://cuehealth.com/products/how-cue-detects-covid-19/. Accessed 22 Sept 2021.

[182] Simplexa^™^ molecular assays. https://www.focusdx.com/product-catalog/simplexa. Accessed 12 Sept 2021.

[183] QuanPLEX. http://www.each-reach.com/platforms-solutions-menu-zh/pcr-platform-menu-zh. Accessed 24 Aug 2021.

[184] Microchip based real-time pcr analyzer ariadna. https://www.lumexinstruments.com/catalog/pcr_analysis/aria_dna.php. Accessed 24 Aug 2021.

[185] Novodiag. https://mobidiag.com/products/novodiag/. Accessed 12 Sept 2021.

[186] cobas^®^ Liat^®^ PCR System. https://diagnostics.roche.com/us/en/products/systems/cobas-liat-system.html. Accessed 24 Aug 2021.

[187] iGeneTec MA3000. http://www.igenetec.com/index.php?c=product&v=list&id=1644. Accessed 24 Aug 2021.

[188] Tsinghua University School of Pharmacy and School of Medicine team jointly developed a nucleic acid test kit, home testing results in 30 minutes. http://www.sps.tsinghua.edu.cn/cn/news/achievement/2020/0529/672.html. Accessed 22 Sept 2021.

[189] Visby medical^TM^. https://www.visbymedical.com/. Accessed 22 Sept 2021.

[190] WizDx^™^ F-150 Real-time PCR System. https://www.wizbiosolution.com/product_view.php?idx=80. Accessed 24 Aug 2021.

[191] Harding-Esch EM, Cousins EC, Chow SL, Phillips LT, Hall CL, Cooper N (2018). A 30-min nucleic acid amplification point-of-care test for genital *Chlamydia trachomatis* infection in women: a prospective, multi-center study of diagnostic accuracy. EBioMedicine.

[192] The binx io. https://mybinxhealth.com/point-of-care. Accessed 22 Sept 2021.

[193] Alere^™^ q A platform to answer global health needs:TB and beyond. https://www.finddx.org/wp-content/uploads/2016/03/FIND-7thSymposium-2014-DuncanBLAIR.pdf. Accessed 12 Sept 2021.

[194] Idyllatm platform. https://www.biocartis.com/en/meet-idylla/idylla-platform. Accessed 12 Sept 2021.

[195] Ichip-400. http://www.bai-care.com/. Accessed 24 Aug 2021.

[196] Huang E, Wang Y, Yang N, Shu B, Zhang G, Liu D (2021). A fully automated microfluidic PCR-array system for rapid detection of multiple respiratory tract infection pathogens. Anal Bioanal Chem.

[197] BD MAX. https://www.bd.com. Accessed 12 Sept 2021.

[198] GeneXpert^®^ infinity systems. https://www.cepheid.com/en_US/systems/GeneXpert-Family-of-Systems/GeneXpert-Infinity. Accessed 22 Sept 2021.

[199] Curetis Unyvero A50. https://curetis.com/products/unyvero-a50-system/. Accessed 12 Sept 2021.

[200] Revogene. https://www.meridianbioscience.com/platform/molecular/revogene/. Accessed 24 Aug 2021.

[201] The ePlex system: the true sample-to-answer solution^®^. https://genmarkdx.com/systems/eplex-system/. Accessed 24 Aug2021.

[202] Verigene. http://www.nanosphere.us/tags/verigene. Accessed 12 Sept 2021.

